# Supplementary material for: Gender differences in clinical presentation and illicit substance use during first episode psychosis: a natural language processing, electronic case register study
Source: BMJ Open. 2021 Apr 20;11(4):e042949. doi: 10.1136/bmjopen-2020-042949 (PMC8061860; doi:10.1136/bmjopen-2020-042949)
Supplement: Supplementary data [file bmjopen-2020-042949supp001.pdf]

Supplementary material

Contents

Table 1: Predictor definitions: self-assigned ethnicity 2

Table 2: Extraction and definition of diagnoses at 12 months 3

Table 3: Manual validation of symptom and substance NLP tools 5

Figure 1: Density plots of substance use by age of onset for four different drugs: amphetamine, cannabis, cocaine and MDMA 7

Table 4: Full table of gender differences in symptom presentation and substance use 8

Table 5: VIF values for each linear model 12

Table 1: Predictor definitions: self-assigned ethnicity

Ethnic group	Self-assigned ethnicity captured in electronic patient records
Asian	Asian or Asian British - Bangladeshi<br>Asian or Asian British - Indian<br>Asian or Asian British - Pakistani<br>Asian or Asian British - Any other Asian background<br>Other Ethnic Groups - Chinese
Black – African	Black or Black British - African
Black – Caribbean	Black or Black British - Caribbean
Black – Other	Black or Black British - Any other Black background<br>Mixed - White and Black African<br>Mixed - White and Black Caribbean
Mixed	Mixed - White and Asian<br>Mixed - Any other mixed background
Other	Other Ethnic Groups - Any other ethnic group
Missing	Not known<br>Not recorded

Irving J, *et al. BMJ Open* 2021; 11:e042949. doi: 10.1136/bmjopen-2020-042949

Table 2: Extraction and definition of diagnoses at 12 months

Diagnosis code	Recorded diagnosis (structured or NLP)
Schizophrenia	'%schizophreni%' or<br>'%scizophreni%' or<br>'%schizotyp%' or<br>'%scizotyp%' or<br>'%f20%' or<br>'%f21%' or<br>'%f22%' or<br>'%f23%' or<br>'%f24%' or<br>'%f28%' or<br>'%f29%'
Schizoaffective disorder	'%schizoaffect%' or<br>'%scizoaffect%' or<br>'%f25%'
Bipolar disorder	('%f30%' or<br>'%f31%' or<br>'%manic%' or<br>'%mania%' or<br>'%bipolar%' or<br>'%bpad%' or<br>'%affective disorder%' or<br>'%mixed affective%') **and**<br>not like '%trichotillomania%' and<br>not like '%kleptomania%'
Psychotic depression	('%psychosis%' or<br>'%psychotic%' or<br>'%with psyc%' or<br>'%f32.3%' or<br>'%f33.3%') **and**<br>('%depress%' or<br>'%f32%' or<br>'%f33%') **and**<br>not like '%without%'
Drug psychosis	('%drug%' or<br>'%alcohol%' or<br>'%opioid%' or<br>'%opiate%' or<br>'%cannabi%' or<br>'%benzo%' or<br>'%hallucinogen%' or<br>'%cocaine%' or<br>'%cannabis%' or<br>'%f10%' or<br>'%f11%' or<br>'%f12%' or<br>'%f13%' or<br>'%f14%' or<br>'%f15%' or<br>'%f16%' or

Irving J, *et al. BMJ Open* 2021; 11:e042949. doi: 10.1136/bmjopen-2020-042949

	'%f17%' or<br>'%f18%' or<br>'%f19%') **and**<br>'%psychotic%'
Other psychosis	All other psychosis mentions not classified above.

Table 3: Manual validation of symptom and substance NLP tools

Symptom	Cohort	Annotations validated (n)	Precision
Aggression	All patients	50	76%
Agitation	All patients	50	82%
Anhedonia	Ever had depression dx	30	87%
Apathy	Ever had depression dx	30	73%
Blunted flat affect	All patients	30	93%
Circumstantial speech	All patients	50	90%
Delusion	All patients	30	87%
Derailment	All patients	50	74%
Disturbed sleep	Ever had depression dx	50	84%
Elation	All patients	30	90%
Emotional withdrawal	All patients	50	64%
Flight of ideas	All patients	50	72%
Formal thought disorder	All patients	50	72%
Grandiosity	All patients	30	97%
Guilt	Ever had depression dx	30	93%
Hallucinations (auditory and visual)	All patients	30	87%
Hallucinations (olfactory, tactile and gustatory)	All patients	50	86%
Helplessness	Ever had depression dx	30	90%
Hopelessness	Ever had depression dx	30	87%
Hostility	All patients	30	87%
Insomnia	Ever had depression dx	50	94%
Irritability	Ever had depression dx	30	93%
Low appetite	Ever had depression dx	30	97%
Low energy	Ever had depression dx	50	76%
Low mood	Ever had depression dx	100	71%
Mood instability	All patients	50	72%
Mutism	All patients	30	93%
Negative symptoms	All patients	30	87%
Paranoia	All patients	50	82%
Persecutory ideation	All patients	30	87%
Poor concentration	Ever had depression dx	50	76%
Poor insight	All patients	30	83%

Poverty of speech	All patients	30	87%
Poverty of thought	All patients	30	83%
Psychomotor retardation	Ever had depression dx	30	90%
Social withdrawal	All patients	30	90%
Tangential speech	All patients	30	97%
Tearfulness	Ever had depression dx	30	99%
Thought block	All patients	30	93%
Weight loss	Ever had depression dx	30	97%
Worthlessness	Ever had depression dx	30	90%
**Substance**	**Cohort**	**Annotations validated (n)**	**Precision**
Amphetamine	All patients	30	90%
Cannabis	All patients	30	93%
Cocaine	All patients	30	97%
MDMA	All patients	30	87%

Irving J, et al. BMJ Open 2021; 11:e042949. doi: 10.1136/bmjopen-2020-042949

Figure 1: Density plots of age at referral by substance use

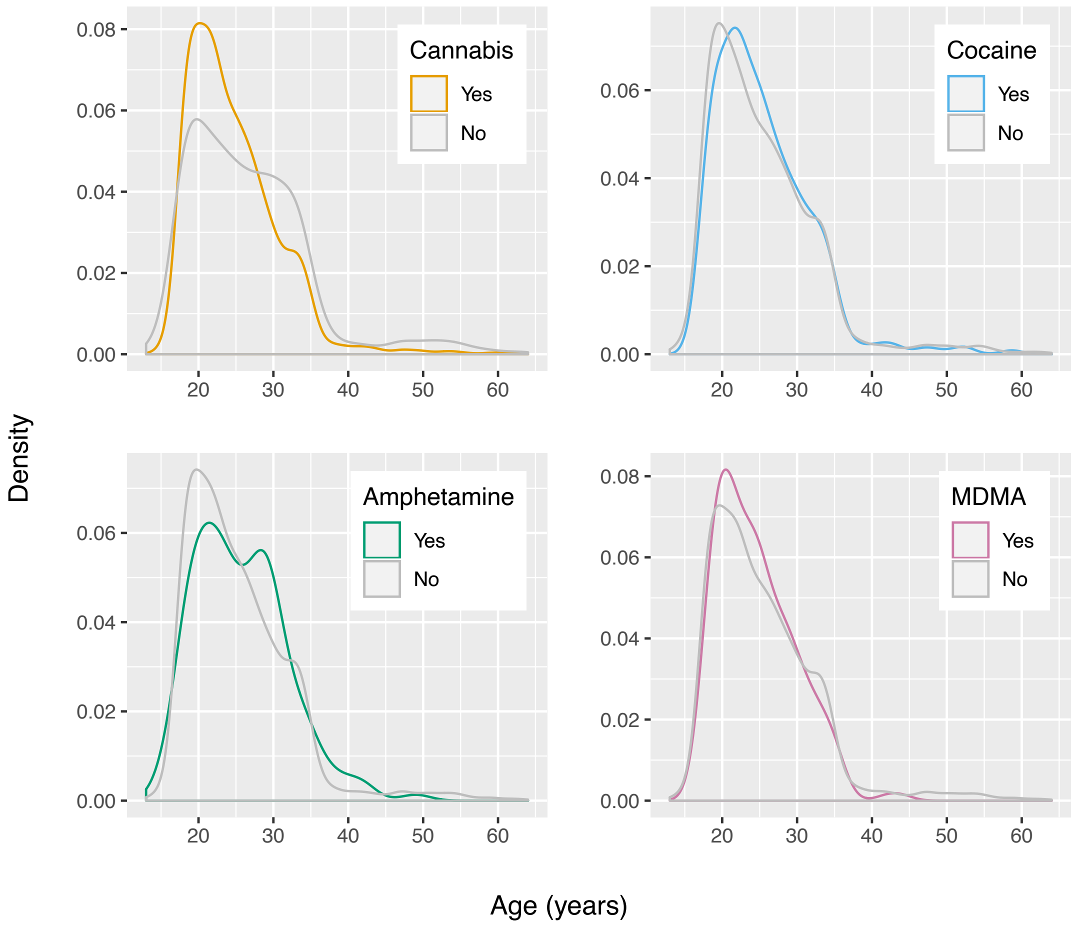

Table 4: Full table of gender differences in symptom presentation and substance use

Symptom/ Substance	N	n (%) female	n (%) male	Unadjusted		Adjusted for age and ethnicity		Adjusted for age, ethnicity and substance use		Adjusted for age, ethnicity, substance use and gender*substance use interaction	
				OR	*P value*[a]	OR	*P value*[a]	OR	*P value*[a]	OR	*P value*[a]
**Positive symptoms**											
Delusions	2584	957 (76.0)	1596 (76.3)	1.01 [0.86 - 1.19]	0.905	1.08 [0.91 - 1.27]	0.537	0.88 [0.74-1.05]	0.278	0.70 [0.54 - 0.91]	0.025*
Hallucinations (auditory and visual)	1384	503 (40.0)	869 (41.5)	1.07 [0.93 - 1.23]	0.43	1.05 [0.91 - 1.21]	0.666	0.98 [0.84-1.13]	0.854	0.97 [0.76 - 1.25]	0.909
Hallucinations (olfactory, tactile and gustatory)	376	162 (12.9)	204 (9.8)	0.73 [0.59 - 0.91]	0.007**	0.74 [0.59 - 0.92]	0.022*	0.71 [0.56-0.89]	.008**	0.76 [0.51 - 1.13]	0.336
Aggression	2318	802 (63.8)	1494 (71.4)	1.42 [1.22 - 1.65]	<0.001***	1.44 [1.24 - 1.67]	<0.001***	1.13 [0.97-1.33]	0.227	1.07 [0.84 - 1.37]	0.737
Agitation	2450	879 (69.9)	1545 (73.9)	1.22 [1.04 - 1.42]	0.017*	1.22 [1.05 - 1.43]	0.031*	0.98 [0.83-1.16]	0.903	1.01 [0.79 - 1.29]	0.996
Hostility	1282	477 (37.9)	801 (38.3)	1.02 [0.88 - 1.17]	0.858	1.03 [0.89 - 1.19]	0.839	0.82 [0.70-0.95]	0.025*	0.78 [0.59 - 1.03]	0.197
Paranoia	3092	1122 (89.2)	1917 (91.6)	1.33 [1.05 - 1.68]	0.025*	1.38 [1.09 - 1.76]	0.021*	1.02 [0.79-1.30]	0.952	0.88 [0.64 - 1.22]	0.640
Persecution ideation	1963	717 (57.0)	1221 (58.4)	1.06 [0.92 - 1.22]	0.495	1.14 [0.98 - 1.31]	0.167	0.98 [0.84-1.14]	0.865	0.89 [0.70 - 1.14]	0.544
**Negative symptoms**											
Blunted flat affect	1045	376 (29.9)	662 (31.6)	1.09 [0.93 - 1.26]	0.355	1.08 [0.93 - 1.26]	0.473	0.99 [0.84-1.16]	0.934		0.352

										0.83 [0.63 - 1.10]	
Emotional withdrawal	1434	520 (41.3)	903 (43.2)	1.08 [0.94 - 1.24]	0.366	1.05 [0.91 - 1.21]	0.668	0.98 [0.85-1.14]	0.896	0.91 [0.71 - 1.16]	0.629
Social withdrawal	647	185 (14.7)	454 (21.7)	1.61 [1.33 - 1.94]	<0.001***	1.56 [1.29 - 1.88]	<0.001***	1.50 [1.23-1.82]	<0.001***	1.43 [1.04 - 1.98]	0.083
Negative symptom (general)	568	150 (11.9)	411 (19.6)	1.81 [1.48 - 2.22]	<0.001***	1.76 [1.43 - 2.16]	<0.001***	1.71 [1.39-2.11]	<0.001***	1.78 [1.26 - 2.51]	0.005**
Poverty of speech	347	102 (8.1)	239 (11.4)	1.46 [1.15 - 1.87]	0.003**	1.45 [1.14 - 1.86]	0.010**	1.43 [1.11-1.86]	0.015*	1.52 [1.01 - 2.29]	0.110
Poverty of thought	187	47 (3.7)	140 (6.7)	1.85 [1.33 - 2.62]	0.001***	1.77 [1.27 - 2.52]	0.004**	1.71 [1.21-2.45]	0.009**	2.01 [1.14 - 3.64]	0.052
Apathy	343	129 (10.3)	208 (9.9)	0.97 [0.77 - 1.22]	0.815	0.97 [0.77 - 1.23]	0.89	0.89 [0.70-1.14]	0.509	0.84 [0.54 - 1.31]	0.650
Mutism	625	272 (21.6)	353 (16.9)	0.74 [0.62 - 0.88]	0.001***	0.72 [0.60 - 0.86]	0.001***	0.66 [0.55-0.80]	<0.001***	0.55 [0.39 - 0.77]	0.002 **
**Mania symptoms**											
Disturbed sleep	2733	1032 (82.0)	1664 (79.4)	0.85 [0.71 - 1.02]	0.098	0.86 [0.72 - 1.03]	0.184	0.73 [0.61 - 0.88]	0.004**	0.62 [0.47 - 0.82]	0.003**
Elation	1060	425 (33.8)	626 (29.9)	0.84 [0.72 - 0.97]	0.026*	0.82 [0.71 - 0.96]	0.033*	0.67 [0.57 - 0.79]	<0.001***	0.62 [0.46 - 0.84]	0.008 **
Grandiosity	1061	351 (27.9)	702 (33.5)	1.31 [1.12 - 1.52]	0.001***	1.33 [1.14 - 1.55]	0.001***	1.12 [0.96 - 1.32]	0.265	1.11 [0.83 - 1.49]	0.660
Insomnia	830	345 (27.4)	479 (22.8)	0.79 [0.67 - 0.92]	0.005**	0.78 [0.66 - 0.92]	0.009**	0.73 [0.62 - 0.87]	0.001***	0.73 [0.55 - 0.98]	0.097
Irritability	2032	754 (59.9)	1262 (60.2)	1.02 [0.88 - 1.17]	0.858	1.02 [0.88 - 1.18]	0.88	0.83 [0.72 - 0.97]	0.045*	0.83 [0.65 - 1.06]	0.278
Mood instability	1763	717 (57.0)	1029 (49.1)	0.73 [0.63 - 0.84]	<0.001***	0.71 [0.61 - 0.81]	<0.001***	<0.58 [0.50 - 0.67]	<0.001***	0.76 [0.60 - 0.97]	0.080
Pressured speech	698	302 (24.0)	388 (18.5)	0.72 [0.61 - 0.85]	<0.001***	0.73 [0.61 - 0.86]	0.001***	0.59 [0.50 - 0.71]	<0.001***	0.68 [0.47 - 0.96]	0.086

**Depressive symptoms**											
Worthless	306	129 (10.3)	171 (8.2)	0.78 [0.61 - 0.99]	0.053	0.78 [0.61 - 0.99]	0.095	0.74 [0.58 - 0.95]	0.045*	0.78 [0.49 - 1.21]	0.447
Anhedonia	546	207 (16.5)	333 (15.9)	0.96 [0.80 - 1.16]	0.746	0.97 [0.80 - 1.18]	0.854	0.95 [0.78-1.16]	0.736	0.78 [0.55 - 1.09]	0.282
Low mood	3087	1162 (92.4)	1874 (89.4)	0.71 [0.55 - 0.91]	0.011*	0.72 [0.56 - 0.93]	0.031*	0.52 [0.40-0.68]	<0.001***	0.51 [0.37 - 0.72]	0.001***
Guilt	1009	426 (33.9)	576 (27.5)	0.74 [0.64 - 0.86]	<.001***	0.74 [0.64 - 0.86]	0.001***	.68 [0.58 - 0.79]	<0.001***	0.68 [0.52 - 0.90]	0.025*
Poor concentration	2110	817 (64.9)	1265 (60.3)	0.83 [0.71 - 0.95]	0.014*	0.80 [0.69 - 0.92]	0.008**	0.73 [0.63 - 0.85]	<0.001***	0.78 [0.61 - 1.00]	0.119
Reduced appetite	1405	593 (47.1)	794 (37.9)	0.69 [0.60 - 0.79]	<0.001***	0.69 [0.60 - 0.79]	<0.001***	0.65 [0.56-0.75]	<0.001***	0.60 [0.47 - 0.77]	<0.000***
Low energy	1137	503 (40.0)	622 (29.7)	0.64 [0.55 - 0.74]	<0.001***	0.64 [0.55 - 0.74]	<0.001***	0.59 [0.51 - 0.69]	<0.001***	0.51 [0.39 - 0.67]	<0.000***
Helpless	262	113 (43.5)	147 (0.7)	0.77 [0.59 - 0.99]	0.053	0.77 [0.59 - 0.99]	0.098	0.76 [0.58 - 1.00]	0.096	0.71 [0.45 - 1.10]	0.274
Hopeless	859	311 (37.0)	529 (25.2)	1.03 [0.88 - 1.21]	0.772	1.04 [0.88 - 1.22]	0.791	1.02 [0.87-1.21]	0.864	1.07 [0.81 - 1.41]	0.810
Psychomotor retardation	357	133 (9.8)	222 (10.6)	1.00 [0.80 - 1.26]	0.971	0.99 [0.79 - 1.25]	0.972	1.01 [0.80-1.29]	0.955	0.94 [0.64 - 1.37]	0.868
Tearful	1723	864 (68.7)	833 (39.7)	0.30 [0.26 - 0.35]	<0.001***	0.30 [0.26 - 0.35]	<0.001***	0.26 [0.22-0.30]	<0.001***	0.30 [0.23 - 0.39]	<0.000***
**Disorganisation**											
Circumstantial speech	573	217 (17.2)	351 (61.8)	0.97 [0.80 - 1.17]	0.774	1.01 [0.84 - 1.22]	0.965	0.88 [0.73 - 1.07]	0.327	1.20 [0.83 - 1.72]	0.515
Derailment of speech	330	118 (9.4)	207 (9.9)	1.06 [0.84 - 1.35]	0.692	1.07 [0.84 - 1.36]	0.757	0.99 [0.78-1.27]	0.97	1.24 [0.80 - 1.92]	0.528
Flight of ideas	561	243 (19.3)	313 (14.9)	0.73 [0.61 - 0.88]	0.002**	0.74 [0.61 - 0.89]	0.005**	0.61 [0.51-0.75]	<0.001***	0.74 [0.50 - 1.09]	0.267

Formal thought disorder	452	169 (13.4)	279 (13.3)	0.99 [0.81 - 1.22]	0.946	1.00 [0.82 - 1.24]	0.985	0.88 [0.71-1.09]	0.368	0.87 [0.57 - 1.30]	0.688
Tangential speech	1181	471 (37.4)	693 (33.0)	0.83 [0.72 - 0.96]	0.015*	0.85 [0.73 - 0.99]	0.079	0.71 [0.60-0.82]	<0.001***	0.81 [0.61 - 1.07]	0.276
Thought block	757	272 (21.6)	475 (22.7)	1.06 [0.90 - 1.26]	0.521	1.04 [0.88 - 1.24]	0.762	0.95 [0.80-1.14]	0.722	1.09 [0.80 - 1.49]	0.751
											**Other**
Poor insight	2200	829 (65.9)	1345 (64.1)	0.93 [0.80 - 1.08]	0.405	0.98 [0.84 - 1.14]	0.883	0.79 [0.67-0.93]	0.011*	0.69 [0.54 - 0.88]	0.011*
**Substance use**											
Cocaine	958	255 (20.3)	685 (32.7)	1.91 [1.63 - 2.26]	<0.001***	1.94 [1.65 - 2.30]	<0.001***	n/a	n/a		n/a
Amphetamine	157	41 (3.3)	114 (5.4)	1.71 [1.20 - 2.49]	0.006**	1.72 [1.20 - 2.51]	0.012*	n/a	n/a		n/a
MDMA	233	79 (6.3)	149 (7.1)	1.14 [0.87 - 1.52]	0.405	1.09 [0.82 - 1.46]	0.699	n/a	n/a		n/a
Cannabis	2223	611 (48.6)	1570 (74.9)	3.18 [2.75 - 3.70]	<0.001***	3.18 [2.74 - 3.71]	<0.001***	n/a	n/a		n/a

Table 5: VIF values for each linear model

Model	Covariate	GVIF	Df	GVIF^(1/(2*Df))
**Appetite**	Gender	1.09	1	1.04
	Age	1.03	1	1.02
	Ethnicity	1.04	5	1.00
	Substance use	1.11	1	1.06
**Energy**	Gender	1.09	1	1.04
	Age	1.03	1	1.02
	Ethnicity	1.04	5	1.00
	Substance use	1.12	1	1.06
**Helpless**	Gender	1.08	1	1.04
	Age	1.03	1	1.02
	Ethnicity	1.04	5	1.00
	Substance use	1.11	1	1.05
**Hopeless**	Gender	1.08	1	1.04
	Age	1.03	1	1.02
	Ethnicity	1.04	5	1.00
	Substance use	1.11	1	1.05
**Psychomotor**	Gender	1.09	1	1.05
	Age	1.03	1	1.02
	Ethnicity	1.05	5	1.00
	Substance use	1.12	1	1.06
**Tearful**	Gender	1.14	1	1.07
	Age	1.03	1	1.02
	Ethnicity	1.04	5	1.00
	Substance use	1.17	1	1.08
**Weight loss**	Gender	1.09	1	1.04
	Age	1.03	1	1.02
	Ethnicity	1.04	5	1.00
	Substance use	1.11	1	1.05
**Worthless**	Gender	1.08	1	1.04
	Age	1.03	1	1.01
	Ethnicity	1.04	5	1.00
	Substance use	1.10	1	1.05
**Suicide**	Gender	1.08	1	1.04
	Age	1.03	1	1.02
	Ethnicity	1.04	5	1.00
	Substance use	1.10	1	1.05
**Anhedonia**	Gender	1.08	1	1.04
	Age	1.03	1	1.02
	Ethnicity	1.04	5	1.00
	Substance use	1.11	1	1.05
**Low Mood**	Gender	1.09	1	1.05
	Age	1.03	1	1.02
	Ethnicity	1.05	5	1.00
	Substance use	1.12	1	1.06
**Guilt**	Gender	1.09	1	1.04
	Age	1.03	1	1.02
	Ethnicity	1.04	5	1.00
	Substance use	1.11	1	1.05
**Concentration**	Gender	1.09	1	1.04
	Age	1.03	1	1.02
	Ethnicity	1.04	5	1.00
	Substance use	1.11	1	1.06
**Mutism**	Gender	1.10	1	1.05
	Age	1.03	1	1.02
	Ethnicity	1.05	5	1.00
	Substance use	1.12	1	1.06
**Circumstantial speech**	Gender	1.07	1	1.04
	Age	1.04	1	1.02
	Ethnicity	1.04	5	1.00
	Substance use	1.11	1	1.05
**Derailment of speech**	Gender	1.07	1	1.04
	Age	1.03	1	1.02
	Ethnicity	1.04	5	1.00
	Substance use	1.10	1	1.05

**Flight of ideas**	Gender	1.08	1	1.04
	Age	1.03	1	1.02
	Ethnicity	1.04	5	1.00
	Substance use	1.10	1	1.05
**Formal thought disorder**	Gender	1.08	1	1.04
	Age	1.03	1	1.02
	Ethnicity	1.04	5	1.00
	Substance use	1.10	1	1.05
**Tangential speech**	Gender	1.09	1	1.04
	Age	1.04	1	1.02
	Ethnicity	1.04	5	1.00
	Substance use	1.13	1	1.06
**Thought block**	Gender	1.08	1	1.04
	Age	1.03	1	1.01
	Ethnicity	1.04	5	1.00
	Substance use	1.10	1	1.05
**Insight**	Gender	1.09	1	1.05
	Age	1.04	1	1.02
	Ethnicity	1.06	5	1.01
	Substance use	1.14	1	1.07
**Disturbed sleep**	Gender	1.09	1	1.05
	Age	1.03	1	1.02
	Ethnicity	1.05	5	1.00
	Substance use	1.12	1	1.06
**Elation**	Gender	1.09	1	1.05
	Age	1.03	1	1.01
	Ethnicity	1.05	5	1.00
	Substance use	1.12	1	1.06
**Grandiosity**	Gender	1.07	1	1.04
	Age	1.03	1	1.02
	Ethnicity	1.05	5	1.00
	Substance use	1.10	1	1.05
**Insomnia**	Gender	1.08	1	1.04
	Age	1.03	1	1.02
	Ethnicity	1.04	5	1.00
	Substance use	1.11	1	1.05
**Irritability**	Gender	1.09	1	1.05
	Age	1.03	1	1.02
	Ethnicity	1.05	5	1.00
	Substance use	1.12	1	1.06
**Apathy**	Gender	1.08	1	1.04
	Age	1.03	1	1.02
	Ethnicity	1.04	5	1.00
	Substance use	1.10	1	1.05
**Blunted flat affect**	Gender	1.08	1	1.04
	Age	1.03	1	1.02
	Ethnicity	1.04	5	1.00
	Substance use	1.11	1	1.05
**Emotional withdrawal**	Gender	1.08	1	1.04
	Age	1.03	1	1.01
	Ethnicity	1.04	5	1.00
	Substance use	1.11	1	1.05
**Negative symptoms (general)**	Gender	1.07	1	1.04
	Age	1.02	1	1.01
	Ethnicity	1.04	5	1.00
	Substance use	1.10	1	1.05
**Poverty of speech**	Gender	1.09	1	1.04
	Age	1.03	1	1.02
	Ethnicity	1.04	5	1.00
	Substance use	1.11	1	1.05
**Poverty of thought**	Gender	1.08	1	1.04
	Age	1.02	1	1.01
	Ethnicity	1.04	5	1.00
	Substance use	1.10	1	1.05
**Social withdrawal**	Gender	1.07	1	1.04
	Age	1.02	1	1.01

	Ethnicity	1.04	5	1.00
	Substance use	1.10	1	1.05
**Aggression**	Gender	1.08	1	1.04
	Age	1.03	1	1.02
	Ethnicity	1.05	5	1.01
	Substance use	1.12	1	1.06
**Agitation**	Gender	1.09	1	1.04
	Age	1.03	1	1.02
	Ethnicity	1.05	5	1.01
	Substance use	1.12	1	1.06
**Delusion**	Gender	1.09	1	1.04
	Age	1.03	1	1.02
	Ethnicity	1.05	5	1.00
	Substance use	1.12	1	1.06
**Hallucinations (auditory and visual)**	Gender	1.08	1	1.04
	Age	1.03	1	1.02
	Ethnicity	1.04	5	1.00
	Substance use	1.11	1	1.05
**Hostility**	Gender	1.09	1	1.04
	Age	1.03	1	1.02
	Ethnicity	1.05	5	1.00
	Substance use	1.12	1	1.06
**Paranoia**	Gender	1.08	1	1.04
	Age	1.03	1	1.01
	Ethnicity	1.04	5	1.00
	Substance use	1.11	1	1.05
**Persecution ideation**	Gender	1.08	1	1.04
	Age	1.04	1	1.02
	Ethnicity	1.05	5	1.00
	Substance use	1.12	1	1.06
**Hallucinations (olfactory, tactile and gustatory)**	Gender	1.08	1	1.04
	Age	1.03	1	1.02
	Ethnicity	1.04	5	1.00
	Substance use	1.11	1	1.05
**Mood instability**	Gender	1.11	1	1.05
	Age	1.03	1	1.02
	Ethnicity	1.04	5	1.00
	Substance use	1.13	1	1.06
**Pressured speech**	Gender	1.09	1	1.04
	Age	1.03	1	1.02
	Ethnicity	1.04	5	1.00
	Substance use	1.11	1	1.06

Irving J, *et al. BMJ Open* 2021; 11:e042949. doi: 10.1136/bmjopen-2020-042949

Table 6: Gender differences in diagnosis at 12 months

Diagnosis at 12 months	N	N (%) Female	N (%) Male	Unadjusted		Adjusted for age and ethnicity	
				OR (95% CI)	*P value*[a]	OR (95% CI)	*P value*[a]
Schizophrenia	1556	519 (41%)	1037 (49%)	*Ref.*	*Ref.*	*Ref.*	*Ref.*
Bipolar disorder	165	89 (7%)	76 (4%)	0.43 [0.31 - 0.59]	<0.001***	0.41 [0.30 - 0.57]	<0.001***
Drug-induced psychosis	112	16 (1%)	96 (5%)	3.00 [1.75 - 5.15]	<0.001***	2.98 [1.73 - 5.12]	<0.001***
Psychotic depression	136	77 (6%)	59 (3%)	0.38 [0.27 - 0.55]	<0.001***	0.39 [0.27 - 0.55]	<0.001***
Schizoaffective	57	32 (3%)	25 (1%)	0.39 [0.23 - 0.66]	<0.001***	0.39 [0.23 - 0.66]	<0.001***
Other psychosis	1324	525 (42%)	799 (38%)	0.76 [0.65 - 0.89]	<0.001***	0.75 [0.64 - 0.88]	0.001

[a] P-values adjusted for multiple testing via FDR

Irving J, *et al. BMJ Open* 2021; 11:e042949. doi: 10.1136/bmjopen-2020-042949
