# Supplementary material for: Gender differences in clinical presentation and illicit substance use during first episode psychosis: a natural language processing, electronic case register study
Source: BMJ Open. 2021 Apr 20;11(4):e042949. doi: 10.1136/bmjopen-2020-042949 (PMC8061860; doi:10.1136/bmjopen-2020-042949)
Supplement: Author's manuscript [file bmjopen-2020-042949.draft_revisions.pdf]

BMJ Open is committed to open peer review. As part of this commitment we make the peer review history of every article we publish publicly available.

When an article is published we post the peer reviewers' comments and the authors' responses online. We also post the versions of the paper that were used during peer review. These are the versions that the peer review comments apply to.

The versions of the paper that follow are the versions that were submitted during the peer review process. They are not the versions of record or the final published versions. They should not be cited or distributed as the published version of this manuscript.

BMJ Open is an open access journal and the full, final, typeset and author-corrected version of record of the manuscript is available on our site with no access controls, subscription charges or pay-per-view fees (http://bmjopen.bmj.com).

If you have any questions on BMJ Open's open peer review process please email info.bmjopen@bmj.com

BMJ Open

Gender differences in clinical presentation and illicit substance use during first episode psychosis: a natural language processing, electronic case register study

Journal:	*BMJ Open*
Manuscript ID	bmjopen-2020-042949
Article Type:	Original research
Date Submitted by the Author:	20-Jul-2020
Complete List of Authors:	Irving, Jessica; Institute of Psychiatry Psychology and Neuroscience, Psychosis Studies<br>Colling, Craig; South London and Maudsley NHS Foundation Trust, Biomedical Research Centre<br>Shetty, Hitesh; South London and Maudsley NHS Foundation Trust, Biomedical Research Centre Nucleus<br>Pritchard, Megan; Institute of Psychiatry Psychology and Neuroscience, Psychological Medicine; South London and Maudsley NHS Foundation Trust, Biomedical Research Centre<br>Stewart, Robert; Institute of Psychiatry Psychology and Neuroscience, Psychological Medicine; South London and Maudsley NHS Foundation Trust, Biomedical Research Centre<br>Fusar-Poli, Paolo; South London and Maudsley NHS Foundation Trust<br>McGuire, Philip; Institute of Psychiatry Psychology and Neuroscience, Psychosis Studies; South London and Maudsley NHS Foundation Trust<br>Patel, Rashmi; Institute of Psychiatry Psychology and Neuroscience, Psychosis Studies; South London and Maudsley NHS Foundation Trust
Keywords:	Schizophrenia & psychotic disorders < PSYCHIATRY, Health informatics < BIOTECHNOLOGY & BIOINFORMATICS, Substance misuse < PSYCHIATRY

SCHOLARONE™
Manuscripts

I, the Submitting Author has the right to grant and does grant on behalf of all authors of the Work (as defined in the below author licence), an exclusive licence and/or a non-exclusive licence for contributions from authors who are: i) UK Crown employees; ii) where BMJ has agreed a CC-BY licence shall apply, and/or iii) in accordance with the terms applicable for US Federal Government officers or employees acting as part of their official duties; on a worldwide, perpetual, irrevocable, royalty-free basis to BMJ Publishing Group Ltd ("BMJ") its licensees and where the relevant Journal is co-owned by BMJ to the co-owners of the Journal, to publish the Work in this journal and any other BMJ products and to exploit all rights, as set out in our licence.

The Submitting Author accepts and understands that any supply made under these terms is made by BMJ to the Submitting Author unless you are acting as an employee on behalf of your employer or a postgraduate student of an affiliated institution which is paying any applicable article publishing charge ("APC") for Open Access articles. Where the Submitting Author wishes to make the Work available on an Open Access basis (and intends to pay the relevant APC), the terms of reuse of such Open Access shall be governed by a Creative Commons licence – details of these licences and which Creative Commons licence will apply to this Work are set out in our licence referred to above.

Other than as permitted in any relevant BMJ Author's Self Archiving Policies, I confirm this Work has not been accepted for publication elsewhere, is not being considered for publication elsewhere and does not duplicate material already published. I confirm all authors consent to publication of this Work and authorise the granting of this licence.

For peer review only

Gender differences in clinical presentation and illicit substance use during first episode psychosis: a natural language processing, electronic case register study

Jessica Irving, MSc[1], Craig Colling, MSc[2], Hitesh Shetty, MSc[2], Megan Pritchard, MSc[1,2], Robert Stewart, PhD, MD[1,2], Paolo Fusar-Poli, PhD, MD[1,2], Philip McGuire, PhD, MD[1,2], Rashmi Patel, PhD, MD[1,2]

1. King's College London (Institute of Psychiatry, Psychology and Neuroscience), London, UK
2. South London and Maudsley NHS Foundation Trust, London, UK

Correspondence to:

Jessica Irving

Tel: +44 (0) 7722555091

E-mail: jessica.irving@kcl.ac.uk

Word count: 3041

Abstract: 274

Total: 3315

Keywords: natural language processing, first episode psychosis, gender differences, substance abuse, electronic case register

For peer review only

Competing interests: All authors have completed the ICMJE uniform disclosure form at www.icmje.org/coi_disclosure.pdf and declare: RS has received research funding from Roche, Janssen, GSK and Takeda. PFP has received grant funds from Lundbeck and honoraria from Lundbeck, Menarini and Angelini outside the current study. RP has received grant funds from Janssen and consultancy fees from Induction Healthcare and Holmusk. The other authors declare no competing interests.

Author contributions: The study was conceived by RP and JI. Data extraction and statistical analysis was performed by JI with support from HS, MP and RP. Reporting of findings were carried out by JI and RP. All authors (JI, CC, HS, MP, RS, PFP, PM and RP) contributed to study design, manuscript preparation and approved the final version.

Ethics approval: The CRIS data resource received ethical approval as an anonymized dataset for secondary analyses from Oxfordshire REC C (Ref: 18/SC/0372).

Source of funding: HS, MP, RS and PM receive funding from the National Institute for Health Research (NIHR) Biomedical Research Centre at South London and Maudsley NHS Foundation Trust and King's College London, which also supports the development and maintenance of the BRC Case Register. RP has received support from a Medical Research Council (MRC) Health Data Research UK Fellowship (MR/S003118/1) and a Starter Grant for Clinical Lecturers (SGL015/1020) supported by the Academy of Medical Sciences, The Wellcome Trust, MRC, British Heart Foundation, Arthritis Research UK, the Royal College of Physicians and Diabetes UK.

Role of funder: The views expressed are those of the authors and not necessarily those of the NHS, the NIHR or the Department of Health. The funders had no role in the design and conduct of the study; collection, management, analysis, and interpretation of the data; preparation, review, or approval of the manuscript; and decision to submit the manuscript for publication.

Data sharing: The data accessed by CRIS remain within an NHS firewall and governance is provided by a patient-led oversight committee. Subject to these conditions, data access is encouraged and those interested should contact RS (robert.stewart@kcl.ac.uk), CRIS academic lead.

For peer review only - http://bmjopen.bmj.com/site/about/guidelines.xhtml

Abstract

Objective: To determine whether gender differences in symptom presentation at first episode of psychosis remain even when controlling for substance use, age and ethnicity, using natural language processing (NLP) applied to electronic health records (EHRs).

Design, Setting, Participants: Data were extracted from EHRs of 3,340 people (62% male) who had presented to the South London and Maudsley (SLaM) NHS Trust with a first episode of psychosis between 1 April 2007 and 31 March 2017. Logistic regression was used to examine gender differences in the presentation of positive, negative, depressive, mania and disorganization symptoms.

Exposure(s) (for observational studies): Gender (male vs female)

Main Outcome(s) and Measure(s): Presence of positive, negative, depressive, mania and disorganization symptoms at initial clinical presentation

Results: Eight symptoms were significantly more prevalent in males (poverty of thought, negative symptoms, social withdrawal, poverty of speech, aggression, grandiosity, paranoia and agitation). Conversely, tearfulness, low energy, reduced appetite, low mood, pressured speech, mood instability, flight of ideas, guilt, mutism, insomnia, poor concentration, tangentiality and elation were more prevalent in females than males. Negative symptoms were more common among males (OR 1.85, 95% CI 1.33 to 2.62) and depressive and manic symptoms more common among females (0.30, 0.26 to 0.35). After adjustment for illicit substance use, the strength of associations between gender and negative, manic and depression symptoms increased, whereas gender differences in aggression, agitation, paranoia and grandiosity became insignificant.

Conclusions: There are clear gender differences in the clinical presentation of FEP. Our findings suggest that gender can have a substantial influence on the nature of clinical presentation in people with psychosis, and that this is only partly explained by exposure to illicit substance use.

For peer review only - http://bmjopen.bmj.com/site/about/guidelines.xhtml

Strengths and limitations

- Our methodology using natural language processing (NLP) on an electronic case register allows us to obtain the largest sample size to date in this field
- This is the first study to control for onset age, ethnicity and substance use in gender differences in symptom presentation in first episode psychosis
- By examining patients at first presentation, our findings represent differences early on in the trajectory of psychosis that are less likely to be affected by treatment.
- NLP techniques are associated with a degree of measurement error, which could impact the recorded frequencies for each symptom
- Symptoms reported in electronic health records might be impacted by gender biases among clinicians

For peer review only

Introduction

The incidence and clinical presentation of first episode psychosis (FEP) varies by gender.[1] Some of these differences, such as earlier age of onset for males, and a bimodal age distribution for females, are well established.[2–4] Others, such as symptoms at FEP presentation, are less well established.

Previous studies report that male patients with FEP present with greater negative and disorganization symptom burden and greater illicit substance use than females.[5] Female patients conversely appear to present with more affective symptoms and parasuicidal behavior than males, with affective symptoms increasing risk for parasuicidal behavior.[6,7] Despite these findings, several studies have failed to find significant clinical differences in symptoms at all.[8,9]

At population level, men show greater risk for substance use than women,[10] and this pattern is especially prominent in psychosis samples.[11–13] Negative symptoms, such as motivational deficits, are common to both psychosis and substance use disorders;[14] increased prevalence of negative symptoms in males could therefore plausibly be associated with illicit substance misuse.[3] However, no published studies have attempted to control for illicit substance use at the point of an emerging psychotic disorder.

Few studies investigating variation in psychotic symptoms at first presentation control for ethnicity. Both psychosis risk and symptomatology show ethnic variability;[1,15–17] furthermore, specific genders and ethnic groups are associated with unique and shared risk factors for psychosis.[18] The way in which these factors might interact in psychosis is unknown.[19]

Quantifying the impact of gender on symptom expression can provide a clue as to the underlying mechanisms. For example, negative symptoms are difficult to treat and correlate with poorer outcomes.[20] The cause of negative symptoms remains unknown, and this is the rate-limiting obstacle to developing new treatments. If these are genuinely more common in men, even factoring in increased substance use in males, it suggests that gender-related biological factors may play a role in their pathophysiology.[3] For example, sex-specific hypothalamic-pituitary-gonadal dysfunction are implicated in psychosis pathophysiology;[21] estrogens are known to modulate the dopaminergic and glutamatergic systems, both of which show aberrant functioning in psychosis.[22] More recently, central nervous system autoimmunity has been implicated in the etiology of psychosis. Around 80% of patients with Anti-N-methyl-d-aspartate (NMDA) receptor encephalitis are female whereas Anti-LGI1 and anti-CASPR2 encephalitis more frequently occur in males.[23] Understanding these gender differences might inform the development of novel, more targeted treatments for psychosis.

We sought to investigate gender differences in psychosis symptoms experienced by those presenting with FEP to a specialized Early Intervention Service using natural language processing (NLP) techniques applied to electronic health record (EHR) data. Natural language processing (NLP) techniques applied to free-text EHR data can automatically classify patients who present with specific symptoms and other characteristics.[24] This approach has previously been used to investigate the association of negative

For peer review only - http://bmjopen.bmj.com/site/about/guidelines.xhtml

symptoms with antipsychotic treatment failure,[25] poor insight as a predictor of service use outcomes[26] and the association of cannabis use with hospital admissions[27] in people with psychotic disorders. Our sample of 3,340 patients represents the largest known sample in this field to date.

For peer review only

For peer review only - http://bmjopen.bmj.com/site/about/guidelines.xhtml

Methods

Reporting

We used the STROBE cross sectional checklist when writing our report. [28]

Study setting

Clinical data were obtained from de-identified EHRs held by the South London and Maudsley (SLaM) NHS Trust using the Clinical Record Interactive Search (CRIS) system. SLaM is one of the largest mental healthcare providers in Europe and implemented a fully electronic health record system from 2006 onwards. The Trust holds records for over 450,000 patients. Its provision includes four early intervention services in its catchment area of four boroughs (Croydon, Lambeth, Lewisham and Southwark) in southeast London with a population of around 1.2 million residents.

As EHRs provided limited structured information on symptomatology and substance use, a suite of natural language processing (NLP) tools has recently been developed within CRIS to allow conversion of unstructured free text, such as that of uploaded attachments and discharge summaries, into structured data. These yield complementary information on diagnoses, symptomatology and other patient characteristics.

The SLaM Biomedical Research Centre Case Register and CRIS have received ethical approval from the Oxfordshire Research Ethics Committee C (18/SC/0372) as an anonymized dataset for mental health research. As CRIS data are de-identified, individual consent was not sought.

Sample

Data were extracted for all individuals aged 16-65 years accepted to Early Intervention Services (EIS) for FEP within the SLaM catchment area (Lambeth Early Onset (LEO) Team, Southwark Team for Early Psychosis (STEP), Lewisham Early Intervention Service, and Croydon Outreach and Assertive Support Team (COAST)) between 1 April 2007 and 31 March 2017. From 2016, the upper age limit of EIS increased from 35 to 65. The original sample contained 3,597 individuals. Individuals with no symptom data (n = 63) and missing ethnicity data (n = 70) were excluded, leaving a sample size of 3464 participants with complete covariate data and at least one documented symptom. Inspection of acceptance date distribution revealed mass uploads of backlogged records on three outlier days in April 2007 for one service; we therefore removed these individuals (n = 114) from analysis as they were not genuine first episodes.

Measures

Demographics

Structured data were extracted on age at accepted referral, gender, and ethnicity. Ethnicity was recoded as Asian, Black – African, Black – Caribbean, Black – Other, Mixed, Other and White (*Supplementary Table 1*).

For peer review only - http://bmjopen.bmj.com/site/about/guidelines.xhtml

Symptoms and substance use

Established CRIS-NLP algorithms were used to extract data for 42 symptom constructs and 4 substance use indicators mentioned in the period three months either side of the date of each patient being accepted to an EIS clinical team. Dichotomous variables were created indicating presence or absence of at least one mention of a given symptom or substance use indicator. We excluded symptoms of low prevalence (less than 0.05% of the sample). Symptoms were classified into the following domains identified in a principal axis factor analysis by Demjaha and colleagues[29] using the approach employed by Jackson *et al* [24] (see Table 2): (1) positive, (2) negative, (3) disorganization, (4) manic and (5) depressive symptoms. *Insight* was assigned to (6) other.

NLP algorithm performance is mainly measured using precision (proportion of relevant instances among retrieved instances) and recall (proportion of relevant instances retrieved from all relevant instances in data). Precision estimates generated through manual validation of each symptom (0.64-0.99, mean = 0.86) and substance (0.87-0.97, mean = 0.92) NLP algorithms are supplied in *Supplementary Table 3*.[24]

Diagnosis

We recorded each patient's ICD-10 diagnosis closest to 12 months from the EIS accepted date. Patient diagnoses were derived from either a structured Primary Diagnosis field or unstructured free text using NLP. Psychosis diagnoses were recoded as bipolar disorder, drug-induced psychosis, schizophrenia, psychotic depression, schizoaffective disorder or other psychosis (*Supplement*).

Statistical analysis

All analyses were conducted using R version 3.6.0. We obtained descriptive statistics for demographic characteristics, diagnosis at 12 months, and symptom and substance use distributions.

Binary logistic regression was used to assess gender differences in symptomatology and substance use. Subsequent multivariable models controlling for age and ethnicity were constructed to examine potential confounding effects in differences in symptoms or substance use. The reference group for ethnicity was 'White', which comprised the sample majority. P-values were adjusted for multiple testing using the Benjamini-Hochberg correction (false discovery rate [FDR])[30] using the *p.adjust* package in R.

The adjusted models were then updated to include illicit substance use as an additional covariate. A dichotomous variable was created to flag any mention of cannabis, cocaine, MDMA or amphetamine as *present/absent*.

Given that *p*-values for male gender increased when controlling for substance use, we ran post-hoc analyses in which we (i) ran diagnostic tests for multicollinearity by calculating the variance inflation factor (VIF) for each predictor in each model, and (ii) built an additional model inclusive of a gender*substance use interaction term.

For peer review only - http://bmjopen.bmj.com/site/about/guidelines.xhtml

Multinomial logistic regression, implemented via the *nnet* package, was used to investigate gender differences in diagnosis at 12 months from the patient's first accepted referral to an EIS. We controlled for age and ethnicity.

Patient and public involvement

Project applications requiring access to CRIS data are subject to approval from the CRIS Oversight committee, which is service user led.

For peer review only

For peer review only - http://bmjopen.bmj.com/site/about/guidelines.xhtml

Results

Table 1 presents sample characteristics. The final sample comprised 3,340 patients of predominantly male gender (n = 2077, 62%). Median (IQR) age was 24 (20-29). White (n = 1037), Black-Other (n = 847) and Black-Caribbean (n = 635) were the most frequently represented ethnic groups.

<< Table 1 >>

Age distribution by gender and by substance use are presented in Figure 1. Number of referrals sharply inclines in the late teenage years for both genders. Both genders experience a sharp peak in incidence in the early twenties which falls at a greater rate for male patients than female patients. Individual density plots showing referral numbers by age stratified by amphetamine, cannabis, cocaine and MDMA use are provided in *Supplementary Figure 1.* The age by cannabis use plot most closely mirrors the distribution of overall substance use shown below. Referrals for cannabis and amphetamine users show inversed patterns around age 30.

<< Figure 1 >>

Figure 2 presents gender differences in symptomatology and substance use obtained via binary logistic regressions before and after adjustment for onset age, ethnicity and illicit substance use. Odds ratios (OR) refer to male gender as the reference group. All values and overall odds ratios for each symptom group are provided in *Supplementary Tables 4* and 5.

Of 42 symptoms investigated, 8 were more prevalent in men, and 14 more prevalent in women before adjustment for age, ethnicity and substance use. Highly significant gender differences ($p \leq 0.001$) existed for poor appetite, low energy, tearfulness, guilt, mutism, grandiosity, negative symptoms, social withdrawal, aggression, mood instability and pressured speech. Symptoms more strongly associated with male gender included (in order of decreasing strength of association) poverty of thought, negative symptoms (general), social withdrawal, poverty of speech, aggression, grandiosity, paranoia and agitation. Male gender was also significantly associated with cannabis, cocaine and amphetamine use.

Female patients were significantly more likely to have depressive and manic symptoms recorded than male patients. In order of decreasing strength, female gender was associated with tearfulness, low energy, reduced appetite, low mood, pressured speech, mood instability, guilt, mutism, insomnia, poor concentration and elation.

Delusions and auditory and visual hallucinations did not differ significantly between genders ($p > 0.05$), but olfactory, tactile and gustatory hallucinations were reported more frequently in female patients. Flight of ideas and tangential speech were more frequently recorded in females than males, but no other disorganization symptoms showed significant gender differences.

<< Figure 2 >>

For peer review only - http://bmjopen.bmj.com/site/about/guidelines.xhtml

For peer review only

All significant differences bar tangential speech remained after adjustment for age and ethnicity (see *Supplementary Table 4*). After further adjustment for illicit substance use, gender differences in aggression, agitation, paranoia and grandiosity became non-significant ($p > 0.05$). Significant differences in negative and depression symptoms remained; these symptoms showed increased log odds. Some previously insignificant gender differences (poor insight, disturbed sleep and irritability) became significantly more likely in females. Elation, flight of ideas and tangential speech increased in significance. Gender differences were driven by individual, rather than broad groups of, symptoms (with the exception of disorganisation; *Supplementary Table 5*). No model variables showed variance inflation factor (VIF) values above 1.08, indicating no issues with multicollinearity (*Supplementary Table 6*). Interaction effects are presented in *Supplementary Table 5*.

There were clear gender differences in diagnosis 12 months from service accepted date, which were robust when adjusting for age and ethnicity (*Supplementary Table 7*). Males were three times more likely to receive a non-schizophrenia, updated diagnosis of drug-induced psychosis than females, and more than half as likely to receive a diagnosis of bipolar disorder than females.

For peer review only - http://bmjopen.bmj.com/site/about/guidelines.xhtml

Discussion

The present study investigated gender differences in symptomatology for 3,340 patients presenting with FEP. We found clear gender differences in reported symptoms, of which almost all remained when controlling for age and ethnicity. However, controlling for illicit substance use resulted in several changes to associations of gender with reported symptoms.

In univariate analyses, most negative symptoms, and use of all substances, were reported more frequently for male patients. Female patients showed more manic (bar grandiosity) and depressive symptoms. Our results are consistent with studies that find more negative symptoms and substance use in male FEP patients, and more affective symptoms in female patients.[5,6,31] Many of these differences are found in the general population, such as increased affective symptoms in women, and substance abuse in men.[10,32] The findings for disorganization symptoms were less clear. Where these existed, they tended towards female patients, in contrast to the findings of Thorup and colleagues.[5]

Strikingly, all significant gender differences (bar tangential speech) remained after controlling for age and ethnicity. This suggests that gender differences in symptoms at presentation are independent of age and ethnicity. To our best knowledge, no studies have accounted for ethnicity in analyses of gender symptom differences,[5,11,12,33] despite previous research indicating a significant association between ethnicity and risk of psychosis.[34] The lack of effect of referral age on gender differences is consistent with other studies' findings.[6,31,33]

After adjusting for illicit substance use, age and ethnicity, negative symptoms were still more prominent in males; manic and depressive symptoms became even more prominent in females. This suggests that gender differences in these three symptom domains reflect genuine sex differences in pathophysiology. Biologically, endocrine variation (particularly hypothalamic-pituitary-gonadal dysfunction in both sexes) has been implicated in gender differences of psychosis presentation. For example, converging epidemiological, clinical and animal research lends support to a neuroprotective effect of estrogens in women as a buffer against illness development and severity.[35] Interestingly, symptom severity appears to increase in the low estrogen phase of the menstrual cycle and during the menopause. Estrogen's neuroprotective effects have been attributed to its effects on the dopaminergic and glutamatergic systems, which are both implicated in psychosis.[21] Anatomically, disrupted sexual dimorphism of several regions in the brain has been repeatedly observed in psychosis.[3] Men and women are also differentially exposed to psychosocial risk factors for psychosis (recently summarized in an umbrella review)[1]. The immune system also varies by gender, and autoimmune central nervous system disorders that show associations with psychosis vary in incidence between men and women.[23]

While referral numbers in cannabis users peak at age 21, those in amphetamine users have bimodal peaks at ages 22 and 29. As our sample already have psychosis at first assessment, any inferences drawn from these findings are speculative. It is possible that patients with cannabis use are presenting with psychosis at a younger age, which is consistently replicated in the literature.[36] This finding could also reflect differential

For peer review only - http://bmjopen.bmj.com/site/about/guidelines.xhtml

age trends for usage of amphetamines and cannabis, with amphetamine more popularly used at a later age than cannabis. Conversely, both drugs might be used at comparable rates by both age groups, with amphetamine use demonstrating a larger effect in older age groups. It could also be that cannabis use confers the biggest immediate risk to the adolescent brain whereas amphetamine use has longer-term effects.

Our findings suggest that substance use plays an important role in clinical presentation of some symptoms, with differential effects between genders. Gender differences in aggression, agitation, paranoia and grandiosity all became insignificant, which might be explained by their associations with illicit substance use. Consistent with previous findings, male FEP patients were far more likely to use cannabis, cocaine and amphetamine than female FEP patients.[32] Comorbid substance use in these populations is associated with medication non-compliance, greater risk of relapse, higher suicidality and poorer outcomes in symptoms and functioning overall.[37–39]

Introduction of illicit substance use as a covariate caused the pattern of observed gender differences to change considerably, often inflating significance. Model VIF values close to 1 discount multicollinearity as a plausible mechanism for inflated probability values. One Danish study has provided initial evidence of a role for substance use in gender differences in clinical presentation in 578 patients assessed five years on from first episode.[5] The authors found significant associations between both male gender and substance abuse for negative and disorganization symptoms; controlling for substance use reduced effect sizes for male gender and removed prior effects of male gender on global assessment of functioning (GAF) symptom score.

In general, the impact of gender on clinical presentation and outcomes in first episode psychosis is under-researched. While 45,944 papers were published on psychotic disorders between 1995 and 2018, only 291 (0.01%) specifically investigated gender differences (Figure 3).

<< Figure 3 >>

This reflects other areas of medical research where the importance of gender differences are increasingly recognized, such as cardiovascular disease,[40] where clinical presentation and symptoms vary by gender leading to underdiagnosis of heart disease and increased mortality in female patients.[41] Our findings highlight the need to investigate the impact of gender differences on clinical presentation and their underlying pathophysiology, which could affect treatment outcomes following onset of FEP.

Strengths and limitations

There are some limitations to our approach. Symptom NLP algorithms vary in precision and recall performance; this variability in NLP accuracy could impact the recorded frequencies of each symptom.[24,27] However, one would expect measurement error to be spread equally across genders. Furthermore, the algorithms cannot be used to infer symptom severity, as patients who are more unwell or represent complex cases typically have more documentation. Therefore gender differences in symptoms may not

For peer review only - http://bmjopen.bmj.com/site/about/guidelines.xhtml

generalize across the range of disease severity. There was no significant difference in document count between genders, therefore this is unlikely to bias results. We had no healthy control group and therefore cannot establish whether gendered symptom differences are specific to FEP or extend to the general population. Given service changes in 2016, there also is only one year of data available for individuals aged 36 and older. Finally, symptoms reported in EHRs might to some extent reflect gender biases in recording or patients' willingness to disclose.

Despite these limitations, our approach also has its strengths. To our knowledge, this is the largest study to investigate gender differences in FEP, and the only study to control for ethnicity and substance use in doing so. By examining patients at first presentation, our findings represent differences early on in the trajectory of psychosis that are less likely to be affected by treatment.

Conclusion

We found that the clinical presentation of FEP varies by gender and that this is only partly explained by exposure to illicit substances. Most research on gender differences in psychosis has focused on candidate pathways to psychosis as a diagnostic construct. Studies are now urgently needed to link biological and psychosocial risk factors to specific symptoms so that novel, more targeted treatments can be developed.

For peer review only

References

1. Radua J, Ramella-Cravaro V, Ioannidis JPA, et al. What causes psychosis? An umbrella review of risk and protective factors. *World Psychiatry*. 2018;17(1):49-66. doi:10.1002/wps.20490

2. Kirkbride JB, Errazuriz A, Croudace TJ, et al. Incidence of Schizophrenia and Other Psychoses in England, 1950–2009: A Systematic Review and Meta-Analyses. Scott JG, ed. *PLoS One*. 2012;7(3):e31660. doi:10.1371/journal.pone.0031660

3. Riecher-Rössler A, Butler S, Kulkarni J. Sex and gender differences in schizophrenic psychoses—a critical review. *Arch Womens Ment Health*. 2018;21(6):627-648. doi:10.1007/s00737-018-0847-9

4. Ochoa S, Usall J, Cobo J, Labad X, Kulkarni J. Gender differences in schizophrenia and first-episode psychosis: a comprehensive literature review. *Schizophr Res Treatment*. 2012;2012:916198. doi:10.1155/2012/916198

5. Thorup A, Albert N, Bertelsen M, et al. Gender differences in first-episode psychosis at 5-year follow-up – two different courses of disease? Results from the OPUS study at 5-year follow-up. *Eur Psychiatry*. 2014;29(1):44-51. doi:10.1016/J.EURPSY.2012.11.005

6. Barajas A, Ochoa S, Obiols JE, Lalucat-Jo L. Gender Differences in Individuals at High-Risk of Psychosis: A Comprehensive Literature Review. *Sci World J*. 2015;2015:1-13. doi:10.1155/2015/430735

7. Tseliou F, Johnson S, Major B, et al. Gender differences in one-year outcomes of first-presentation psychosis patients in inner-city UK Early Intervention Services. *Early Interv Psychiatry*. 2017;11(3):215-223. doi:10.1111/eip.12235

8. Larsen TK, McGlashan TH, Johannessen JO, Vibe-Hansen L. First-episode Schizophrenia: II. Premorbid Patterns by Gender. *Schizophr Bull*. 1996;22(2):257-269. doi:10.1093/schbul/22.2.257

9. Hayashi N, Igarashi Y, Yamashina M, Suda K. *Is There a Gender Difference in a Factorial Structure of the Positive and Negative Syndrome Scale? A Test by Structural Equation Modeling*.; 2002. www.karger.comwww.karger.com/journals/psp. Accessed June 3, 2019.

10. Seedat S, Scott KM, Angermeyer MC, et al. Cross-national associations between gender and mental disorders in the World Health Organization World Mental Health Surveys. *Arch Gen Psychiatry*. 2009. doi:10.1001/archgenpsychiatry.2009.36

11. Cotton SM, Lambert M, Schimmelmann BG, et al. Gender differences in premorbid, entry, treatment, and outcome characteristics in a treated epidemiological sample of 661 patients with first episode psychosis. *Schizophr Res*. 2009;114(1-3):17-24. doi:10.1016/J.SCHRES.2009.07.002

12. Køster A, Lajer M, Lindhardt A, Rosenbaum B. Gender differences in first episode psychosis. *Soc Psychiatry Psychiatr Epidemiol*. 2008;43(12):940-946. doi:10.1007/s00127-008-0384-3

13. Talamo A, Centorrino F, Tondo L, Dimitri A, Hennen J, Baldessarini RJ. Comorbid substance-use in schizophrenia: Relation to positive and negative symptoms. *Schizophr Res*. 2006. doi:10.1016/j.schres.2006.04.004

14. Solowij N, Michie PT. Cannabis and cognitive dysfunction: Parallels with endophenotypes of schizophrenia? *J Psychiatry Neurosci*. 2007.

15. Heuvelman H, Nazroo J, Rai D. Investigating ethnic variations in reporting of psychotic symptoms: a multiple-

For peer review only - http://bmjopen.bmj.com/site/about/guidelines.xhtml

group confirmatory factor analysis of the Psychosis Screening Questionnaire. *Psychol Med*. 2018;48(16):2757-2765. doi:10.1017/S0033291718000399

16. Fusar-Poli P, Tantardini M, De Simone S, et al. Deconstructing vulnerability for psychosis: Meta-analysis of environmental risk factors for psychosis in subjects at ultra high-risk. *Eur Psychiatry*. 2017;40:65-75. doi:10.1016/j.eurpsy.2016.09.003

17. Oliver D, Reilly TJ, Baccaredda Boy O, et al. What Causes the Onset of Psychosis in Individuals at Clinical High Risk? A Meta-analysis of Risk and Protective Factors. *Schizophr Bull*. June 2019. doi:10.1093/schbul/sbz039

18. Mann F, Fisher HL, Major B, et al. Ethnic variations in compulsory detention and hospital admission for psychosis across four UK Early Intervention Services. *BMC Psychiatry*. 2014. doi:10.1186/s12916-014-0181-7

19. Hankivsky O. Women's health, men's health, and gender and health: Implications of intersectionality. *Soc Sci Med*. 2012. doi:10.1016/j.socscimed.2011.11.029

20. Downs J, Dean H, Lechler S, et al. Negative Symptoms in Early-Onset Psychosis and Their Association With Antipsychotic Treatment Failure. *Schizophr Bull*. 2019;45(1):69-79. doi:10.1093/schbul/sbx197

21. Riecher-Rössler A. Oestrogens, prolactin, hypothalamic-pituitary-gonadal axis, and schizophrenic psychoses. *The Lancet Psychiatry*. 2017. doi:10.1016/S2215-0366(16)30379-0

22. Riecher-Rössler A, Kulkarni J. Estrogens and Gonadal Function in Schizophrenia and Related Psychoses. In: Springer, Berlin, Heidelberg; 2010:155-171. doi:10.1007/7854_2010_100

23. Gold SM, Willing A, Leypoldt F, Paul F, Friese MA. Sex differences in autoimmune disorders of the central nervous system. *Semin Immunopathol*. 2019;41(2):177-188. doi:10.1007/s00281-018-0723-8

24. Jackson RG, Patel R, Jayatilleke N, et al. Natural language processing to extract symptoms of severe mental illness from clinical text: the Clinical Record Interactive Search Comprehensive Data Extraction (CRIS-CODE) project. *BMJ Open*. 2017;7(1):e012012. doi:10.1136/bmjopen-2016-012012

25. Downs J, Dean H, Lechler S, et al. Negative Symptoms in Early-Onset Psychosis and Their Association With Antipsychotic Treatment Failure. *Schizophr Bull*. January 2018. doi:10.1093/schbul/sbx197

26. Ramu N, Kolliakou A, Sanyal J, Patel R, Stewart R. Recorded poor insight as a predictor of service use outcomes: Cohort study of patients with first-episode psychosis in a large mental healthcare database. *BMJ Open*. 2019. doi:10.1136/bmjopen-2019-028929

27. Patel R, Jayatilleke N, Broadbent M, et al. Negative symptoms in schizophrenia: a study in a large clinical sample of patients using a novel automated method. *BMJ Open*. 2015;5(9):e007619. doi:10.1136/bmjopen-2015-007619

28. von Elm E, Altman DG, Egger M, Pocock SJ, Gøtzsche PC, Vandenbroucke JP. The strengthening the reporting of observational studies in epidemiology (STROBE) statement: Guidelines for reporting observational studies. *Int J Surg*. 2014. doi:10.1016/j.ijsu.2014.07.013

29. Demjaha A, Morgan K, Morgan C, et al. Combining dimensional and categorical representation of psychosis: The way forward for DSM-V and ICD-11? *Psychol Med*. 2009. doi:10.1017/S0033291709990651

30. Benjamini Y, Hochberg Y. Controlling the False Discovery Rate: A Practical and Powerful Approach to Multiple

For peer review only - http://bmjopen.bmj.com/site/about/guidelines.xhtml

Testing. *J R Stat Soc Ser B*. 1995. doi:10.1111/j.2517-6161.1995.tb02031.x

31. Køster A, Lajer M, Lindhardt A, Rosenbaum B. Gender differences in first episode psychosis. *Soc Psychiatry Psychiatr Epidemiol*. 2008;43(12):940-946. doi:10.1007/s00127-008-0384-3

32. Larsen TK, Melle I, Auestad B, et al. Substance abuse in first-episode non-affective psychosis. *Schizophr Res*. 2006. doi:10.1016/j.schres.2006.07.018

33. Thorup A, Petersen L, Jeppesen P, et al. Gender differences in young adults with first-episode schizophrenia spectrum disorders at baseline in the Danish OPUS study. *J Nerv Ment Dis*. 2007;195(5):396-405. doi:10.1097/01.nmd.0000253784.59708.dd

34. Boydell J, van Os J, McKenzie K, et al. Incidence of schizophrenia in ethnic minorities in London: ecological study into interactions with environment. *BMJ*. 2001;323(7325):1336-1338. doi:10.1136/bmj.323.7325.1336

35. Gogos A, Sbisa AM, Sun J, Gibbons A, Udawela M, Dean B. A Role for Estrogen in Schizophrenia: Clinical and Preclinical Findings. *Int J Endocrinol*. 2015;2015:615356. doi:10.1155/2015/615356

36. Large M, Sharma S, Compton MT, Slade T, Nielssen O. Cannabis Use and Earlier Onset of Psychosis. *Arch Gen Psychiatry*. 2011;68(6):555. doi:10.1001/archgenpsychiatry.2011.5

37. Lambert M, Conus P, Lubman DI, et al. The impact of substance use disorders on clinical outcome in 643 patients with first-episode psychosis. *Acta Psychiatr Scand*. 2005. doi:10.1111/j.1600-0447.2005.00554.x

38. Cantor-Graae E, Nordström LG, McNeil TF. Substance abuse in schizophrenia: A review of the literature and a study of correlates in Sweden. *Schizophr Res*. 2001. doi:10.1016/S0920-9964(00)00114-6

39. Sorbara F, Liraud F, Assens F, Abalan F, Verdoux H. Substance use and the course of early psychosis: A 2-year follow-up of first-admitted subjects. *Eur Psychiatry*. 2003. doi:10.1016/S0924-9338(03)00027-0

40. Leonard EA, Marshall RJ. Cardiovascular Disease in Women. *Prim Care - Clin Off Pract*. 2018. doi:10.1016/j.pop.2017.10.004

41. Mosca L, Barrett-Connor E, Kass Wenger N. Sex/gender differences in cardiovascular disease prevention: What a difference a decade makes. *Circulation*. 2011. doi:10.1161/CIRCULATIONAHA.110.968792

For peer review only

For peer review only - http://bmjopen.bmj.com/site/about/guidelines.xhtml

Table 1: Sample characteristics

Characteristic	n	Male	Female
N (%)	3350	2092 (62%)	1258 (38%)
Age at referral (Median, IQR)	24 (20-29)	23 (20-28)	25 (21-30)
Ethnicity (N, %)			
Asian	234	144 (7%)	90 (7%)
Black African	635	383 (30%)	252 (20%)
Black Caribbean	216	123 (6%)	93 (7%)
Black Other	847	533 (3%)	314 (25%)
White	1037	652 (49%)	385 (31%)
Other	381	257 (12%)	124 (10%)
Early Intervention Team			
Lambeth	1084	683 (33%)	401 (32%)
Southwark	826	520 (25%)	306 (24%)
Lewisham	678	400 (19%)	278 (22%)
Croydon	761	495 (24%)	266 (21%)

For peer review only

For peer review only - http://bmjopen.bmj.com/site/about/guidelines.xhtml

Figure 1. Density plots of age at referral by gender and by substance use

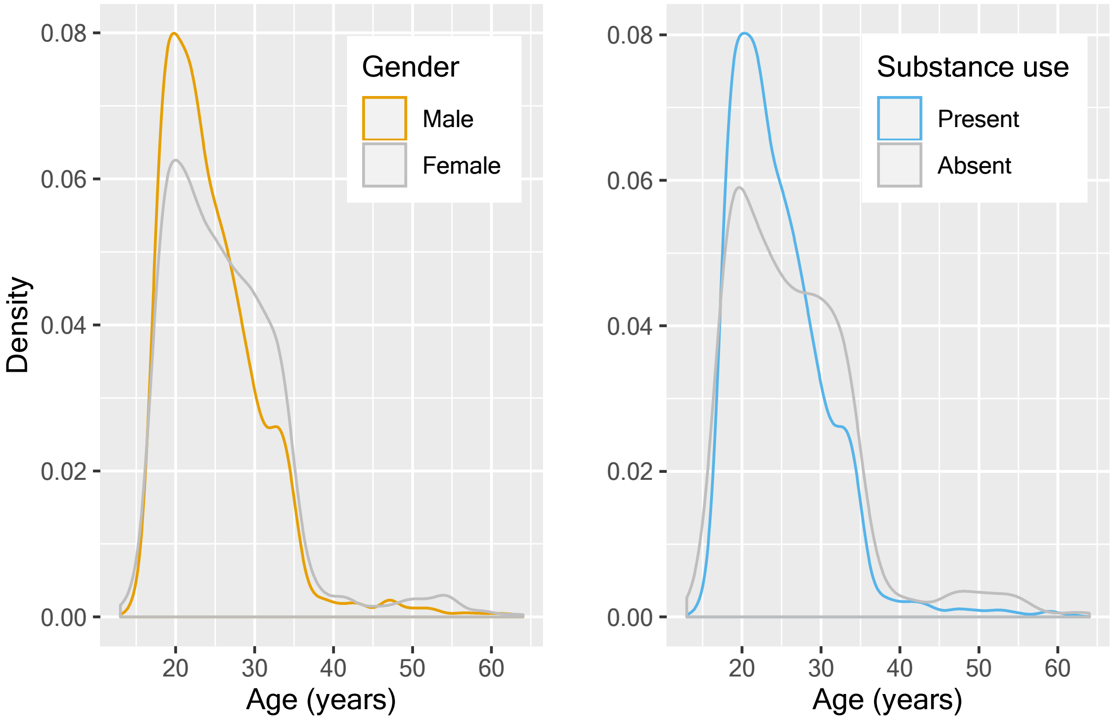

Figure 1 legend: These density plots present age at admission by gender (left) and substance use (right). Density plots – a variation of the histogram – present the distribution of numerical data but with kernel smoothing to reduce the effect of noise in the data, thereby creating a smoother line. The peaks of a density plot indicate where values are concentrated. Both genders experience a sharp peak in incidence in the early twenties, which falls at a greater rate for male patients than female patients. This peak is mirrored in the substance use plot (right), with FEP admissions for substance users more concentrated in the early twenties before sharply declining compared with non-substance users.

For peer review only - http://bmjopen.bmj.com/site/about/guidelines.xhtml

Figure 2: Forest plots presenting odds ratios before and after adjustment for age of onset, ethnicity and illicit substance use

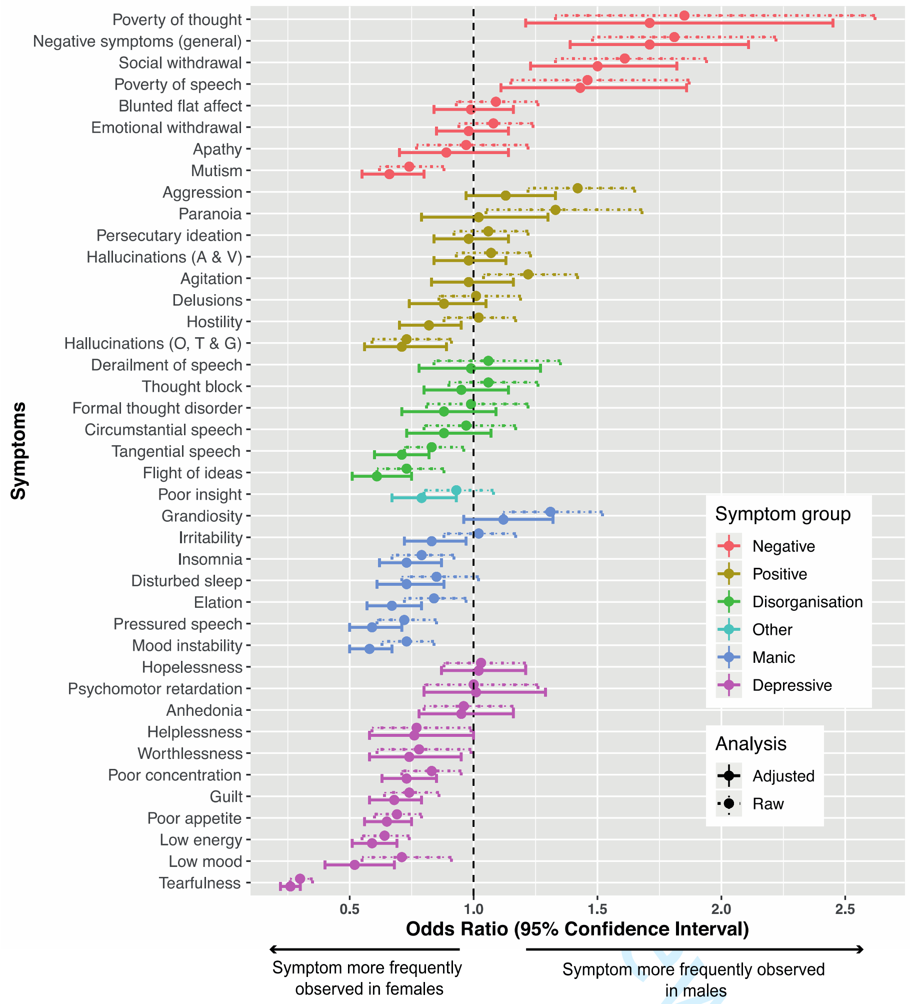

Figure 2 legend: Odds ratios greater than 1 indicate that a symptom is more likely to be observed in males than females, and vice versa for odds ratios less than 1. Dashed lines indicate odds ratios and confidence intervals *before* adjustment for covariates, with solid lines indicating estimates *after* adjustment.

For peer review only - http://bmjopen.bmj.com/site/about/guidelines.xhtml

Figure 3: Research into the role of gender in psychotic disorders between 1998-2018

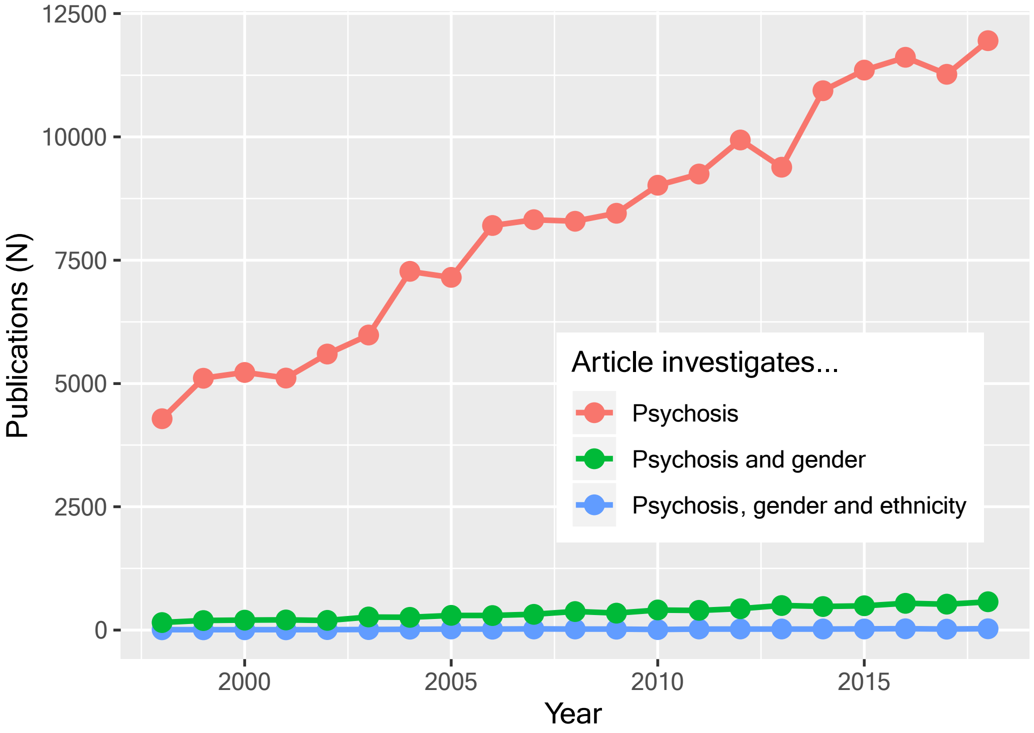

Source: Web of Science accessed January 2020

For peer review only - http://bmjopen.bmj.com/site/about/guidelines.xhtml

Supplementary material

Contents

Table 1: Predictor definitions: self-assigned ethnicity 2
Table 2: Extraction and definition of diagnoses at 12 months 3
Table 3: Manual validation of symptom and substance NLP tools 5
Figure 1: Density plots of age at referral by substance use 8
Table 4: Full table of gender differences in symptom presentation and substance use 9
Table 5: Overall odds ratios for membership of each symptom group 15
Table 6: Variance inflation factor (VIF) values for each linear model 16
Table 7: Gender differences in diagnosis at 12 months 21

For peer review only - http://bmjopen.bmj.com/site/about/guidelines.xhtml

Table 1: Predictor definitions: self-assigned ethnicity

**Ethnic group**	**Self-assigned ethnicity captured in electronic patient records**
Asian	Asian or Asian British - Bangladeshi<br>Asian or Asian British - Indian<br>Asian or Asian British - Pakistani<br>Asian or Asian British - Any other Asian background<br>Other Ethnic Groups - Chinese
Black – African	Black or Black British - African
Black – Caribbean	Black or Black British - Caribbean
Black – Other	Black or Black British - Any other Black background<br>Mixed - White and Black African<br>Mixed - White and Black Caribbean
Mixed	Mixed - White and Asian<br>Mixed - Any other mixed background
Other	Other Ethnic Groups - Any other ethnic group
Missing	Not known<br>Not recorded

For peer review only

Table 2: Extraction and definition of diagnoses at 12 months

**Diagnosis code**	**Recorded diagnosis (structured or NLP)**
Schizophrenia	'%schizophreni%' or<br>'%scizophreni%' or<br>'%schizotyp%' or<br>'%scizotyp%' or<br>'%f20%' or<br>'%f21%' or<br>'%f22%' or<br>'%f23%' or<br>'%f24%' or<br>'%f28%' or<br>'%f29%'
Schizoaffective disorder	'%schizoaffect%' or<br>'%scizoaffect%' or<br>'%f25%'
Bipolar disorder	('%f30%' or<br>'%f31%' or<br>'%manic%' or<br>'%mania%' or<br>'%bipolar%' or<br>'%bpad%' or<br>'%affective disorder%' or<br>'%mixed affective%') **and**<br>not like '%trichotillomania%' and<br>not like '%kleptomania%'
Psychotic depression	('%psychosis%' or<br>'%psychotic%' or<br>'%with psyc%' or<br>'%f32.3%' or<br>'%f33.3%') **and**<br>('%depress%' or

For peer review only - http://bmjopen.bmj.com/site/about/guidelines.xhtml

	'%f32%' or<br>'%f33%') **and**<br>not like '%without%'
Drug psychosis	('%drug%' or<br>'%alcohol%' or<br>'%opioid%' or<br>'%opiate%' or<br>'%cannabi%' or<br>'%benzo%' or<br>'%hallucinogen%' or<br>'%cocaine%' or<br>'%cannabis%' or<br>'%f10%' or<br>'%f11%' or<br>'%f12%' or<br>'%f13%' or<br>'%f14%' or<br>'%f15%' or<br>'%f16%' or<br>'%f17%' or<br>'%f18%' or<br>'%f19%') **and**<br>'%psychotic%'
Other psychosis	All other psychosis mentions not classified above.

For peer review only - http://bmjopen.bmj.com/site/about/guidelines.xhtml

Table 3: Manual validation of symptom and substance NLP tools

Symptom	Cohort	Annotations validated (n)	Precision
Aggression	All patients	50	76%
Agitation	All patients	50	82%
Anhedonia	Ever had depression dx	30	87%
Apathy	Ever had depression dx	30	73%
Blunted flat affect	All patients	30	93%
Circumstantial speech	All patients	50	90%
Delusion	All patients	30	87%
Derailment	All patients	50	74%
Disturbed sleep	Ever had depression dx	50	84%
Elation	All patients	30	90%
Emotional withdrawal	All patients	50	64%
Flight of ideas	All patients	50	72%
Formal thought disorder	All patients	50	72%
Grandiosity	All patients	30	97%
Guilt	Ever had depression dx	30	93%
Hallucinations (auditory and visual)	All patients	30	87%
Hallucinations (olfactory, tactile and gustatory)	All patients	50	86%
Helplessness	Ever had depression dx	30	90%
Hopelessness	Ever had depression dx	30	87%
Hostility	All patients	30	87%
Insomnia	Ever had depression dx	50	94%

For peer review only - http://bmjopen.bmj.com/site/about/guidelines.xhtml

Irritability	Ever had depression dx	30	93%
Low appetite	Ever had depression dx	30	97%
Low energy	Ever had depression dx	50	76%
Low mood	Ever had depression dx	100	71%
Mood instability	All patients	50	72%
Mutism	All patients	30	93%
Negative symptoms	All patients	30	87%
Paranoia	All patients	50	82%
Persecutory ideation	All patients	30	87%
Poor concentration	Ever had depression dx	50	76%
Poor insight	All patients	30	83%
Poverty of speech	All patients	30	87%
Poverty of thought	All patients	30	83%
Psychomotor retardation	Ever had depression dx	30	90%
Social withdrawal	All patients	30	90%
Tangential speech	All patients	30	97%
Tearfulness	Ever had depression dx	30	99%
Thought block	All patients	30	93%
Weight loss	Ever had depression dx	30	97%
Worthlessness	Ever had depression dx	30	90%
**Substance**	**Cohort**	**Annotations validated (n)**	**Precision**
Amphetamine	All patients	30	90%
Cannabis	All patients	30	93%

For peer review only - http://bmjopen.bmj.com/site/about/guidelines.xhtml

Cocaine	All patients	30	97%
MDMA	All patients	30	87%

For peer review only

For peer review only - http://bmjopen.bmj.com/site/about/guidelines.xhtml

Figure 1: Density plots of age at referral by substance use

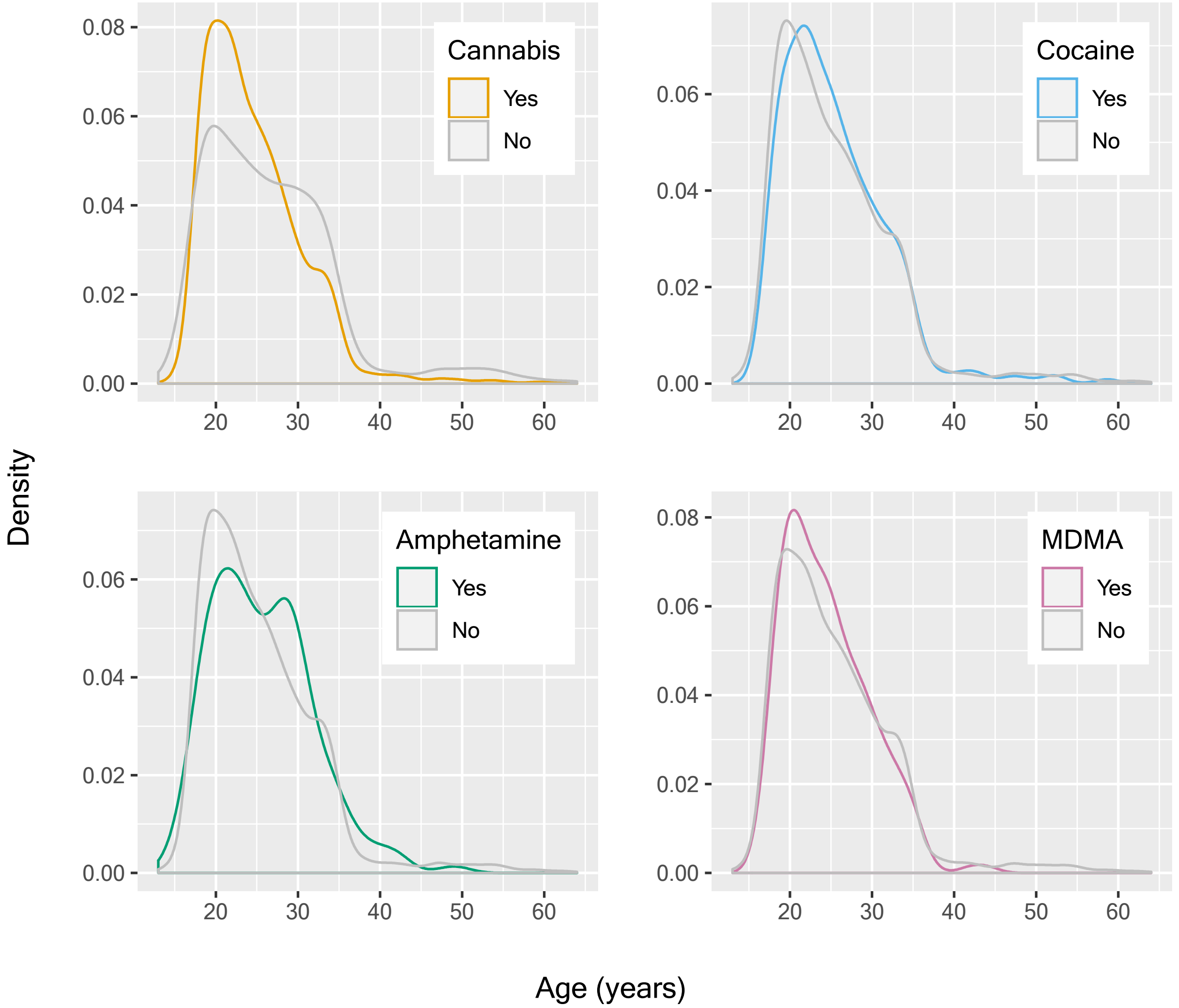

Figure legend: Density plots – a variation of the histogram – present the distribution of numerical data but with kernel smoothing to reduce the effect of noise in the data, thereby creating a smoother line. The peaks of a density plot indicate where values are concentrated. The age by cannabis use plot most closely mirrors the distribution of overall substance use shown in the main text. Referrals for cannabis and amphetamine users show inversed patterns around age 30. There is no real difference in age at admission between cocaine users and non-cocaine users, and only a small difference for MDMA users versus non-users.

For peer review only - http://bmjopen.bmj.com/site/about/guidelines.xhtml

Table 4: Full table of gender differences in symptom presentation and substance use

**Symptom/ Substance**	**N**	**n (%) female**	**n (%) male**	**Unadjusted**		**Adjusted for age and ethnicity**		**Adjusted for age, ethnicity and substance use**		**Adjusted for age, ethnicity, substance use and gender*substance use interaction**	
				**OR**	***P value***[a]	**OR**	***P value***[a]	**OR**	***P value***[a]	**OR**	***P value***[a]
**Positive symptoms**											
Delusions	2584	957 (76.0)	1596 (76.3)	1.01 [0.86 - 1.19]	0.905	1.08 [0.91 - 1.27]	0.537	0.88 [0.74-1.05]	0.278	0.70 [0.54 - 0.91]	0.025*
Hallucinations (auditory and visual)	1384	503 (40.0)	869 (41.5)	1.07 [0.93 - 1.23]	0.43	1.05 [0.91 - 1.21]	0.666	0.98 [0.84-1.13]	0.854	0.97 [0.76 - 1.25]	0.909
Hallucinations (olfactory, tactile and gustatory)	376	162 (12.9)	204 (9.8)	0.73 [0.59 - 0.91]	0.007**	0.74 [0.59 - 0.92]	0.022*	0.71 [0.56-0.89]	.008**	0.76 [0.51 - 1.13]	0.336
Aggression	2318	802 (63.8)	1494 (71.4)	1.42 [1.22 - 1.65]	<0.001***	1.44 [1.24 - 1.67]	<0.001***	1.13 [0.97-1.33]	0.227	1.07 [0.84 - 1.37]	0.737

For peer review only

For peer review only - http://bmjopen.bmj.com/site/about/guidelines.xhtml

Agitation	2450	879 (69.9)	1545 (73.9)	1.22 [1.04 - 1.42]	0.017*	1.22 [1.05 - 1.43]	0.031*	0.98 [0.83-1.16]	0.903	1.01 [0.79 - 1.29]	0.996
Hostility	1282	477 (37.9)	801 (38.3)	1.02 [0.88 - 1.17]	0.858	1.03 [0.89 - 1.19]	0.839	0.82 [0.70-0.95]	0.025*	0.78 [0.59 - 1.03]	0.197
Paranoia	3092	1122 (89.2)	1917 (91.6)	1.33 [1.05 - 1.68]	0.025*	1.38 [1.09 - 1.76]	0.021*	1.02 [0.79-1.30]	0.952	0.88 [0.64 - 1.22]	0.640
Persecution ideation	1963	717 (57.0)	1221 (58.4)	1.06 [0.92 - 1.22]	0.495	1.14 [0.98 - 1.31]	0.167	0.98 [0.84-1.14]	0.865	0.89 [0.70 - 1.14]	0.544
**Negative symptoms**											
Blunted flat affect	1045	376 (29.9)	662 (31.6)	1.09 [0.93 - 1.26]	0.355	1.08 [0.93 - 1.26]	0.473	0.99 [0.84-1.16]	0.934	0.83 [0.63 - 1.10]	0.352
Emotional withdrawal	1434	520 (41.3)	903 (43.2)	1.08 [0.94 - 1.24]	0.366	1.05 [0.91 - 1.21]	0.668	0.98 [0.85-1.14]	0.896	0.91 [0.71 - 1.16]	0.629
Social withdrawal	647	185 (14.7)	454 (21.7)	1.61 [1.33 - 1.94]	<0.001***	1.56 [1.29 - 1.88]	<0.001***	1.50 [1.23-1.82]	<0.001***	1.43 [1.04 - 1.98]	0.083

For peer review only - http://bmjopen.bmj.com/site/about/guidelines.xhtml

Negative symptom (general)	568	150 (11.9)	411 (19.6)	1.81 [1.48 - 2.22]	<0.001***	1.76 [1.43 - 2.16]	<0.001***	1.71 [1.39-2.11]	<0.001***	1.78 [1.26 - 2.51]	0.005**
Poverty of speech	347	102 (8.1)	239 (11.4)	1.46 [1.15 - 1.87]	0.003**	1.45 [1.14 - 1.86]	0.010**	1.43 [1.11-1.86]	0.015*	1.52 [1.01 - 2.29]	0.110
Poverty of thought	187	47 (3.7)	140 (6.7)	1.85 [1.33 - 2.62]	0.001***	1.77 [1.27 - 2.52]	0.004**	1.71 [1.21-2.45]	0.009**	2.01 [1.14 - 3.64]	0.052
Apathy	343	129 (10.3)	208 (9.9)	0.97 [0.77 - 1.22]	0.815	0.97 [0.77 - 1.23]	0.89	0.89 [0.70-1.14]	0.509	0.84 [0.54 - 1.31]	0.650
Mutism	625	272 (21.6)	353 (16.9)	0.74 [0.62 - 0.88]	0.001***	0.72 [0.60 - 0.86]	0.001***	0.66 [0.55-0.80]	<0.001***	0.55 [0.39 - 0.77]	0.002**
**Mania symptoms**											
Disturbed sleep	2733	1032 (82.0)	1664 (79.4)	0.85 [0.71 - 1.02]	0.098	0.86 [0.72 - 1.03]	0.184	0.73 [0.61 - 0.88]	0.004**	0.62 [0.47 - 0.82]	0.003**
Elation	1060	425 (33.8)	626 (29.9)	0.84 [0.72 - 0.97]	0.026*	0.82 [0.71 - 0.96]	0.033*	0.67 [0.57 - 0.79]	<0.001***	0.62 [0.46 - 0.84]	0.008**

For peer review only

Grandiosity	1061	351 (27.9)	702 (33.5)	1.31 [1.12 - 1.52]	0.001***	1.33 [1.14 - 1.55]	0.001***	1.12 [0.96 - 1.32]	0.265	1.11 [0.83 - 1.49]	0.660
Insomnia	830	345 (27.4)	479 (22.8)	0.79 [0.67 - 0.92]	0.005**	0.78 [0.66 - 0.92]	0.009**	0.73 [0.62 - 0.87]	0.001***	0.73 [0.55 - 0.98]	0.097
Irritability	2032	754 (59.9)	1262 (60.2)	1.02 [0.88 - 1.17]	0.858	1.02 [0.88 - 1.18]	0.88	0.83 [0.72 - 0.97]	0.045*	0.83 [0.65 - 1.06]	0.278
Mood instability	1763	717 (57.0)	1029 (49.1)	0.73 [0.63 - 0.84]	<0.001***	0.71 [0.61 - 0.81]	<0.001***	0.58 [0.50 - 0.67]	<0.001***	0.76 [0.60 - 0.97]	0.080
Pressured speech	698	302 (24.0)	388 (18.5)	0.72 [0.61 - 0.85]	<0.001***	0.73 [0.61 - 0.86]	0.001***	0.59 [0.50 - 0.71]	<0.001***	0.68 [0.47 - 0.96]	0.086
**Depressive symptoms**											
Worthless	306	129 (10.3)	171 (8.2)	0.78 [0.61 - 0.99]	0.053	0.78 [0.61 - 0.99]	0.095	0.74 [0.58 - 0.95]	0.045*	0.78 [0.49 - 1.21]	0.447
Anhedonia	546	207 (16.5)	333 (15.9)	0.96 [0.80 - 1.16]	0.746	0.97 [0.80 - 1.18]	0.854	0.95 [0.78-1.16]	0.736	0.78 [0.55 - 1.09]	0.282

For peer review only - http://bmjopen.bmj.com/site/about/guidelines.xhtml

Low mood	3087	1162 (92.4)	1874 (89.4)	0.71 [0.55 - 0.91]	0.011*	0.72 [0.56 - 0.93]	0.031*	0.52 [0.40-0.68]	<0.001***	0.51 [0.37 - 0.72]	0.001***
Guilt	1009	426 (33.9)	576 (27.5)	0.74 [0.64 - 0.86]	<.001***	0.74 [0.64 - 0.86]	0.001***	0.68 [0.58 - 0.79]	<0.001***	0.68 [0.52 - 0.90]	0.025*
Poor concentration	2110	817 (64.9)	1265 (60.3)	0.83 [0.71 - 0.95]	0.014*	0.80 [0.69 - 0.92]	0.008**	0.73 [0.63 - 0.85]	<0.001***	0.78 [0.61 - 1.00]	0.119
Reduced appetite	1405	593 (47.1)	794 (37.9)	0.69 [0.60 - 0.79]	<0.001***	0.69 [0.60 - 0.79]	<0.001***	0.65 [0.56-0.75]	<0.001***	0.60 [0.47 - 0.77]	<0.001***
Low energy	1137	503 (40.0)	622 (29.7)	0.64 [0.55 - 0.74]	<0.001***	0.64 [0.55 - 0.74]	<0.001***	0.59 [0.51 - 0.69]	<0.001***	0.51 [0.39 - 0.67]	<0.001***
Helpless	262	113 (43.5)	147 (0.7)	0.77 [0.59 - 0.99]	0.053	0.77 [0.59 - 0.99]	0.098	0.76 [0.58 - 1.00]	0.096	0.71 [0.45 - 1.10]	0.274
Hopeless	859	311 (37.0)	529 (25.2)	1.03 [0.88 - 1.21]	0.772	1.04 [0.88 - 1.22]	0.791	1.02 [0.87-1.21]	0.864	1.07 [0.81 - 1.41]	0.810
Psychomotor retardation	357	133 (9.8)	222 (10.6)	1.00 [0.80 -	0.971	0.99 [0.79 - 1.25]	0.972	1.01 [0.80-1.29]	0.955	0.94 [0.64 - 1.37]	0.868

				1.26]							
Tearful	1723	864 (68.7)	833 (39.7)	0.30 [0.26 - 0.35]	<0.001***	0.30 [0.26 - 0.35]	<0.001***	0.26 [0.22-0.30]	<0.001***	0.30 [0.23 - 0.39]	<0.001***
**Disorganisation**											
Circumstantial speech	573	217 (17.2)	351 (61.8)	0.97 [0.80 - 1.17]	0.774	1.01 [0.84 - 1.22]	0.965	0.88 [0.73 - 1.07]	0.327	1.20 [0.83 - 1.72]	0.515
Derailment of speech	330	118 (9.4)	207 (9.9)	1.06 [0.84 - 1.35]	0.692	1.07 [0.84 - 1.36]	0.757	0.99 [0.78-1.27]	0.97	1.24 [0.80 - 1.92]	0.528
Flight of ideas	561	243 (19.3)	313 (14.9)	0.73 [0.61 - 0.88]	0.002**	0.74 [0.61 - 0.89]	0.005**	0.61 [0.51-0.75]	<0.001***	0.74 [0.50 - 1.09]	0.267
Formal thought disorder	452	169 (13.4)	279 (13.3)	0.99 [0.81 - 1.22]	0.946	1.00 [0.82 - 1.24]	0.985	0.88 [0.71-1.09]	0.368	0.87 [0.57 - 1.30]	0.688
Tangential speech	1181	471 (37.4)	693 (33.0)	0.83 [0.72 - 0.96]	0.015*	0.85 [0.73 - 0.99]	0.079	0.71 [0.60-0.82]	<0.001***	0.81 [0.61 - 1.07]	0.276
Thought block	757	272 (21.6)	475 (22.7)	1.06 [0.90 -	0.521	1.04 [0.88 - 1.24]	0.762	0.95 [0.80-1.14]	0.722	1.09 [0.80 - 1.49]	0.751

For peer review only

For peer review only - http://bmjopen.bmj.com/site/about/guidelines.xhtml

				1.26]							
**Other**											
Poor insight	2200	829 (65.9)	1345 (64.1)	0.93 [0.80 - 1.08]	0.405	0.98 [0.84 - 1.14]	0.883	0.79 [0.67-0.93]	0.011*	0.69 [0.54 - 0.88]	0.011*
**Substance use**											
Cocaine	958	255 (20.3)	685 (32.7)	1.91 [1.63 - 2.26]	<0.001***	1.94 [1.65 - 2.30]	<0.001***	n/a	n/a		n/a
Amphetamine	157	41 (3.3)	114 (5.4)	1.71 [1.20 - 2.49]	0.006**	1.72 [1.20 - 2.51]	0.012*	n/a	n/a		n/a
MDMA	233	79 (6.3)	149 (7.1)	1.14 [0.87 - 1.52]	0.405	1.09 [0.82 - 1.46]	0.699	n/a	n/a		n/a
Cannabis	2223	611 (48.6)	1570 (74.9)	3.18 [2.75 - 3.70]	<0.001***	3.18 [2.74 - 3.71]	<0.001***	n/a	n/a		n/a

Odds ratios greater than 1 indicate that a symptom is more likely to be observed in males than females, and vice versa for odds ratios less than 1.
[a] All p-values have been adjusted for multiple testing via FDR.
*** P ≤ 0.001
** P ≤ 0.01
* P ≤ 0.05

Table 5: Overall odds ratios for membership of each symptom group

For peer review only - http://bmjopen.bmj.com/site/about/guidelines.xhtml

Symptom group	Odds ratio [95% CI]	FDR-adjusted p-value
Negative	0.97 (0.79 – 1.17)	0.860
Positive	1.39 (0.59 – 3.07)	0.609
Disorganisation	0.71 (0.59 – 0.87)	0.004**
Manic	0.64 (0.40 – 0.99)	0.149
Depressive	0.65 (0.35 – 1.13)	0.273

*** P ≤ 0.001
** P ≤ 0.01
* P ≤ 0.05

For peer review only

Table 6: Variance inflation factor (VIF) values for each linear model

Model	Covariate	GVIF	Df	GVIF^(1/(2*Df))
**Appetite**	Gender	1.09	1	1.04
	Age	1.03	1	1.02
	Ethnicity	1.04	5	1.00
	Substance use	1.11	1	1.06
**Energy**	Gender	1.09	1	1.04
	Age	1.03	1	1.02
	Ethnicity	1.04	5	1.00
	Substance use	1.12	1	1.06
**Helpless**	Gender	1.08	1	1.04
	Age	1.03	1	1.02
	Ethnicity	1.04	5	1.00
	Substance use	1.11	1	1.05
**Hopeless**	Gender	1.08	1	1.04
	Age	1.03	1	1.02
	Ethnicity	1.04	5	1.00
	Substance use	1.11	1	1.05
**Psychomotor**	Gender	1.09	1	1.05
	Age	1.03	1	1.02
	Ethnicity	1.05	5	1.00
	Substance use	1.12	1	1.06
**Tearful**	Gender	1.14	1	1.07
	Age	1.03	1	1.02
	Ethnicity	1.04	5	1.00

For peer review only - http://bmjopen.bmj.com/site/about/guidelines.xhtml

	Substance use	1.17	1	1.08
**Weight loss**	Gender	1.09	1	1.04
	Age	1.03	1	1.02
	Ethnicity	1.04	5	1.00
	Substance use	1.11	1	1.05
**Worthless**	Gender	1.08	1	1.04
	Age	1.03	1	1.01
	Ethnicity	1.04	5	1.00
	Substance use	1.10	1	1.05
**Suicide**	Gender	1.08	1	1.04
	Age	1.03	1	1.02
	Ethnicity	1.04	5	1.00
	Substance use	1.10	1	1.05
**Anhedonia**	Gender	1.08	1	1.04
	Age	1.03	1	1.02
	Ethnicity	1.04	5	1.00
	Substance use	1.11	1	1.05
**Low Mood**	Gender	1.09	1	1.05
	Age	1.03	1	1.02
	Ethnicity	1.05	5	1.00
	Substance use	1.12	1	1.06
**Guilt**	Gender	1.09	1	1.04
	Age	1.03	1	1.02
	Ethnicity	1.04	5	1.00
	Substance use	1.11	1	1.05
**Concentration**	Gender	1.09	1	1.04
	Age	1.03	1	1.02
	Ethnicity	1.04	5	1.00
	Substance use	1.11	1	1.06
**Mutism**	Gender	1.10	1	1.05
	Age	1.03	1	1.02
	Ethnicity	1.05	5	1.00
	Substance use	1.12	1	1.06
**Circumstantial speech**	Gender	1.07	1	1.04
	Age	1.04	1	1.02
	Ethnicity	1.04	5	1.00
	Substance use	1.11	1	1.05
**Derailment of speech**	Gender	1.07	1	1.04
	Age	1.03	1	1.02
	Ethnicity	1.04	5	1.00
	Substance use	1.10	1	1.05

For peer review only

**Flight of ideas**	Gender	1.08	1	1.04
	Age	1.03	1	1.02
	Ethnicity	1.04	5	1.00
	Substance use	1.10	1	1.05
**Formal thought disorder**	Gender	1.08	1	1.04
	Age	1.03	1	1.02
	Ethnicity	1.04	5	1.00
	Substance use	1.10	1	1.05
**Tangential speech**	Gender	1.09	1	1.04
	Age	1.04	1	1.02
	Ethnicity	1.04	5	1.00
	Substance use	1.13	1	1.06
**Thought block**	Gender	1.08	1	1.04
	Age	1.03	1	1.01
	Ethnicity	1.04	5	1.00
	Substance use	1.10	1	1.05
**Insight**	Gender	1.09	1	1.05
	Age	1.04	1	1.02
	Ethnicity	1.06	5	1.01
	Substance use	1.14	1	1.07
**Disturbed sleep**	Gender	1.09	1	1.05
	Age	1.03	1	1.02
	Ethnicity	1.05	5	1.00
	Substance use	1.12	1	1.06
**Elation**	Gender	1.09	1	1.05
	Age	1.03	1	1.01
	Ethnicity	1.05	5	1.00
	Substance use	1.12	1	1.06
**Grandiosity**	Gender	1.07	1	1.04
	Age	1.03	1	1.02
	Ethnicity	1.05	5	1.00
	Substance use	1.10	1	1.05
**Insomnia**	Gender	1.08	1	1.04
	Age	1.03	1	1.02
	Ethnicity	1.04	5	1.00
	Substance use	1.11	1	1.05
**Irritability**	Gender	1.09	1	1.05
	Age	1.03	1	1.02
	Ethnicity	1.05	5	1.00
	Substance use	1.12	1	1.06

For peer review only - http://bmjopen.bmj.com/site/about/guidelines.xhtml

**Apathy**	Gender	1.08	1	1.04
	Age	1.03	1	1.02
	Ethnicity	1.04	5	1.00
	Substance use	1.10	1	1.05
**Blunted flat affect**	Gender	1.08	1	1.04
	Age	1.03	1	1.02
	Ethnicity	1.04	5	1.00
	Substance use	1.11	1	1.05
**Emotional withdrawal**	Gender	1.08	1	1.04
	Age	1.03	1	1.01
	Ethnicity	1.04	5	1.00
	Substance use	1.11	1	1.05
**Negative symptoms (general)**	Gender	1.07	1	1.04
	Age	1.02	1	1.01
	Ethnicity	1.04	5	1.00
	Substance use	1.10	1	1.05
**Poverty of speech**	Gender	1.09	1	1.04
	Age	1.03	1	1.02
	Ethnicity	1.04	5	1.00
	Substance use	1.11	1	1.05
**Poverty of thought**	Gender	1.08	1	1.04
	Age	1.02	1	1.01
	Ethnicity	1.04	5	1.00
	Substance use	1.10	1	1.05
**Social withdrawal**	Gender	1.07	1	1.04
	Age	1.02	1	1.01
	Ethnicity	1.04	5	1.00
	Substance use	1.10	1	1.05
**Aggression**	Gender	1.08	1	1.04
	Age	1.03	1	1.02
	Ethnicity	1.05	5	1.01
	Substance use	1.12	1	1.06
**Agitation**	Gender	1.09	1	1.04
	Age	1.03	1	1.02
	Ethnicity	1.05	5	1.01
	Substance use	1.12	1	1.06
**Delusion**	Gender	1.09	1	1.04
	Age	1.03	1	1.02
	Ethnicity	1.05	5	1.00
	Substance use	1.12	1	1.06

For peer review only

**Hallucinations (auditory and visual)**	Gender	1.08	1	1.04
	Age	1.03	1	1.02
	Ethnicity	1.04	5	1.00
	Substance use	1.11	1	1.05
**Hostility**	Gender	1.09	1	1.04
	Age	1.03	1	1.02
	Ethnicity	1.05	5	1.00
	Substance use	1.12	1	1.06
**Paranoia**	Gender	1.08	1	1.04
	Age	1.03	1	1.01
	Ethnicity	1.04	5	1.00
	Substance use	1.11	1	1.05
**Persecution ideation**	Gender	1.08	1	1.04
	Age	1.04	1	1.02
	Ethnicity	1.05	5	1.00
	Substance use	1.12	1	1.06
**Hallucinations (olfactory, tactile and gustatory)**	Gender	1.08	1	1.04
	Age	1.03	1	1.02
	Ethnicity	1.04	5	1.00
	Substance use	1.11	1	1.05
**Mood instability**	Gender	1.11	1	1.05
	Age	1.03	1	1.02
	Ethnicity	1.04	5	1.00
	Substance use	1.13	1	1.06
**Pressured speech**	Gender	1.09	1	1.04
	Age	1.03	1	1.02
	Ethnicity	1.04	5	1.00
	Substance use	1.11	1	1.06

Variance inflation factor (VIF) is a measure of multicollinearity among multiple regression variables. High VIF values (over X) indicate high collinearity between variables. The closer to 1, the lower the collinearity. No variables used in our regression models yielded VIF values over 1.08, indicating no issues with multicollinearity.

For peer review only

Table 7: Gender differences in diagnosis at 12 months

Diagnosis at 12 months	N	N (%) Female	N (%) Male	Unadjusted		Adjusted for age and ethnicity	
				OR (95% CI)	P value[a]	OR (95% CI)	P value[a]
Schizophrenia	1556	519 (41%)	1037 (49%)	*Ref.*	*Ref.*	*Ref.*	*Ref.*
Bipolar disorder	165	89 (7%)	76 (4%)	0.43 [0.31 - 0.59]	<0.001***	0.41 [0.30 - 0.57]	<0.001***
Drug-induced psychosis	112	16 (1%)	96 (5%)	3.00 [1.75 - 5.15]	<0.001***	2.98 [1.73 - 5.12]	<0.001***
Psychotic depression	136	77 (6%)	59 (3%)	0.38 [0.27 - 0.55]	<0.001***	0.39 [0.27 - 0.55]	<0.001***
Schizoaffective	57	32 (3%)	25 (1%)	0.39 [0.23 - 0.66]	<0.001***	0.39 [0.23 - 0.66]	<0.001***
Other psychosis	1324	525 (42%)	799 (38%)	0.76 [0.65 - 0.89]	<0.001***	0.75 [0.64 - 0.88]	0.001

[a] P-values adjusted for multiple testing via FDR

*** P ≤ 0.001

** P ≤ 0.01

* P ≤ 0.05

For peer review only - http://bmjopen.bmj.com/site/about/guidelines.xhtml

Reporting checklist for cross sectional study.

Based on the STROBE cross sectional guidelines.

Instructions to authors

Complete this checklist by entering the page numbers from your manuscript where readers will find each of the items listed below.

Your article may not currently address all the items on the checklist. Please modify your text to include the missing information. If you are certain that an item does not apply, please write "n/a" and provide a short explanation.

Upload your completed checklist as an extra file when you submit to a journal.

In your methods section, say that you used the STROBE cross sectionalreporting guidelines, and cite them as:

von Elm E, Altman DG, Egger M, Pocock SJ, Gotzsche PC, Vandenbroucke JP. The Strengthening the Reporting of Observational Studies in Epidemiology (STROBE) Statement: guidelines for reporting observational studies.

		Reporting Item	Page Number
**Title and abstract**			
Title	#1a	Indicate the study's design with a commonly used term in the title or the abstract	1
Abstract	#1b	Provide in the abstract an informative and balanced summary of what was done and what was found	4
**Introduction**			
Background / rationale	#2	Explain the scientific background and rationale for the investigation being reported	5
Objectives	#3	State specific objectives, including any prespecified hypotheses	5
**Methods**			
Study design	#4	Present key elements of study design early in the paper	7

For peer review only

Setting	#5	Describe the setting, locations, and relevant dates, including periods of recruitment, exposure, follow-up, and data collection	7
Eligibility criteria	#6a	Give the eligibility criteria, and the sources and methods of selection of participants.	7
	#7	Clearly define all outcomes, exposures, predictors, potential confounders, and effect modifiers. Give diagnostic criteria, if applicable	7-8
Data sources / measurement	#8	For each variable of interest give sources of data and details of methods of assessment (measurement). Describe comparability of assessment methods if there is more than one group. Give information separately for for exposed and unexposed groups if applicable.	7-8
Bias	#9	Describe any efforts to address potential sources of bias	8
Study size	#10	Explain how the study size was arrived at	7
Quantitative variables	#11	Explain how quantitative variables were handled in the analyses. If applicable, describe which groupings were chosen, and why	8
Statistical methods	#12a	Describe all statistical methods, including those used to control for confounding	8-9
Statistical methods	#12b	Describe any methods used to examine subgroups and interactions	8-9
Statistical methods	#12c	Explain how missing data were addressed	7
Statistical methods	#12d	If applicable, describe analytical methods taking account of sampling strategy	n/a
Statistical methods	#12e	Describe any sensitivity analyses	n/a
**Results**			
Participants	#13a	Report numbers of individuals at each stage of study—eg numbers potentially eligible, examined for eligibility, confirmed eligible, included in the study, completing follow-up, and analysed. Give information separately for for exposed and unexposed groups if applicable.	7, 10

For peer review only

Participants	#13b	Give reasons for non-participation at each stage	7
Participants	#13c	Consider use of a flow diagram	n/a
Descriptive data	#14a	Give characteristics of study participants (eg demographic, clinical, social) and information on exposures and potential confounders. Give information separately for exposed and unexposed groups if applicable.	10
Descriptive data	#14b	Indicate number of participants with missing data for each variable of interest	7
Outcome data	#15	Report numbers of outcome events or summary measures. Give information separately for exposed and unexposed groups if applicable.	8
Main results	#16a	Give unadjusted estimates and, if applicable, confounder-adjusted estimates and their precision (eg, 95% confidence interval). Make clear which confounders were adjusted for and why they were included	10-11
Main results	#16b	Report category boundaries when continuous variables were categorized	n/a
Main results	#16c	If relevant, consider translating estimates of relative risk into absolute risk for a meaningful time period	n/a
Other analyses	#17	Report other analyses done—e.g., analyses of subgroups and interactions, and sensitivity analyses	11
**Discussion**			
Key results	#18	Summarise key results with reference to study objectives	12
Limitations	#19	Discuss limitations of the study, taking into account sources of potential bias or imprecision. Discuss both direction and magnitude of any potential bias.	13
Interpretation	#20	Give a cautious overall interpretation considering objectives, limitations, multiplicity of analyses, results from similar studies, and other relevant evidence.	13-14
Generalisability	#21	Discuss the generalisability (external validity) of the study results	14

For peer review only

**Other Information**			
Funding	#22	Give the source of funding and the role of the funders for the present study and, if applicable, for the original study on which the present article is based	2

The STROBE checklist is distributed under the terms of the Creative Commons Attribution License CC-BY.
This checklist was completed on 14. July 2020 using https://www.goodreports.org/, a tool made by the EQUATOR Network in collaboration with Penelope.ai

For peer review only

BMJ Open

Gender differences in clinical presentation and illicit substance use during first episode psychosis: a natural language processing, electronic case register study

Journal:	*BMJ Open*
Manuscript ID	bmjopen-2020-042949.R1
Article Type:	Original research
Date Submitted by the Author:	09-Mar-2021

Complete List of Authors:	Irving, Jessica; Institute of Psychiatry Psychology and Neuroscience, Psychosis Studies<br>Colling, Craig; South London and Maudsley NHS Foundation Trust, Biomedical Research Centre<br>Shetty, Hitesh; South London and Maudsley NHS Foundation Trust, Biomedical Research Centre Nucleus<br>Pritchard, Megan; Institute of Psychiatry Psychology and Neuroscience, Psychological Medicine; South London and Maudsley NHS Foundation Trust, Biomedical Research Centre<br>Stewart, Robert; Institute of Psychiatry Psychology and Neuroscience, Psychological Medicine; South London and Maudsley NHS Foundation Trust, Biomedical Research Centre<br>Fusar-Poli, Paolo; South London and Maudsley NHS Foundation Trust<br>McGuire, Philip; Institute of Psychiatry Psychology and Neuroscience, Psychosis Studies; South London and Maudsley NHS Foundation Trust<br>Patel, Rashmi; Institute of Psychiatry Psychology and Neuroscience, Psychosis Studies; South London and Maudsley NHS Foundation Trust

**Primary Subject Heading**:	Mental health
Secondary Subject Heading:	Health informatics, Epidemiology
Keywords:	Schizophrenia & psychotic disorders < PSYCHIATRY, Health informatics < BIOTECHNOLOGY & BIOINFORMATICS, Substance misuse < PSYCHIATRY

SCHOLARONE™
Manuscripts

I, the Submitting Author has the right to grant and does grant on behalf of all authors of the Work (as defined in the below author licence), an exclusive licence and/or a non-exclusive licence for contributions from authors who are: i) UK Crown employees; ii) where BMJ has agreed a CC-BY licence shall apply, and/or iii) in accordance with the terms applicable for US Federal Government officers or employees acting as part of their official duties; on a worldwide, perpetual, irrevocable, royalty-free basis to BMJ Publishing Group Ltd ("BMJ") its licensees and where the relevant Journal is co-owned by BMJ to the co-owners of the Journal, to publish the Work in this journal and any other BMJ products and to exploit all rights, as set out in our licence.

The Submitting Author accepts and understands that any supply made under these terms is made by BMJ to the Submitting Author unless you are acting as an employee on behalf of your employer or a postgraduate student of an affiliated institution which is paying any applicable article publishing charge ("APC") for Open Access articles. Where the Submitting Author wishes to make the Work available on an Open Access basis (and intends to pay the relevant APC), the terms of reuse of such Open Access shall be governed by a Creative Commons licence – details of these licences and which Creative Commons licence will apply to this Work are set out in our licence referred to above.

Other than as permitted in any relevant BMJ Author's Self Archiving Policies, I confirm this Work has not been accepted for publication elsewhere, is not being considered for publication elsewhere and does not duplicate material already published. I confirm all authors consent to publication of this Work and authorise the granting of this licence.

For peer review only

Gender differences in clinical presentation and illicit substance use during first episode psychosis: a natural language processing, electronic case register study

Jessica Irving, MSc[1], Craig Colling, MSc[2], Hitesh Shetty, MSc[2], Megan Pritchard, MSc[1,2], Robert Stewart, PhD, MD[1,2], Paolo Fusar-Poli, PhD, MD[1,2], Philip McGuire, PhD, MD[1,2], Rashmi Patel, PhD, MD[1,2]

1. King's College London (Institute of Psychiatry, Psychology and Neuroscience), London, UK
2. South London and Maudsley NHS Foundation Trust, London, UK

Correspondence to:

Jessica Irving

Tel: +44 (0) 7722555091

E-mail: jessica.irving@kcl.ac.uk

Word count: 3312

Abstract: 274

Total: 3586

Keywords: natural language processing, first episode psychosis, gender differences, substance abuse, electronic case register

For peer review only

For peer review only - http://bmjopen.bmj.com/site/about/guidelines.xhtml

Competing interests: All authors have completed the ICMJE uniform disclosure form at www.icmje.org/coi_disclosure.pdf and declare: RS has received research funding from Roche, Janssen, GSK and Takeda. PFP has received grant funds from Lundbeck and honoraria from Lundbeck, Menarini and Angelini outside the current study. RP has received grant funds from Janssen and consultancy fees from Induction Healthcare and Holmusk. The other authors declare no competing interests.

Author contributions: The study was conceived by RP and JI. Data extraction and statistical analysis was performed by JI with support from HS, MP and RP. Reporting of findings were carried out by JI and RP. All authors (JI, CC, HS, MP, RS, PFP, PM and RP) contributed to study design, manuscript preparation and approved the final version.

Ethics approval: The CRIS data resource received ethical approval as an anonymized dataset for secondary analyses from Oxfordshire REC C (Ref: 18/SC/0372).

Source of funding: HS, MP, RS and PM receive funding from the National Institute for Health Research (NIHR) Biomedical Research Centre at South London and Maudsley NHS Foundation Trust and King's College London, which also supports the development and maintenance of the BRC Case Register. RP has received support from a Medical Research Council (MRC) Health Data Research UK Fellowship (MR/S003118/1) and a Starter Grant for Clinical Lecturers (SGL015/1020) supported by the Academy of Medical Sciences, The Wellcome Trust, MRC, British Heart Foundation, Arthritis Research UK, the Royal College of Physicians and Diabetes UK.

Role of funder: The views expressed are those of the authors and not necessarily those of the NHS, the NIHR or the Department of Health. The funders had no role in the design and conduct of the study; collection, management, analysis, and interpretation of the data; preparation, review, or approval of the manuscript; and decision to submit the manuscript for publication.

Data sharing: The data accessed by CRIS remain within an NHS firewall and governance is provided by a patient-led oversight committee. Subject to these conditions, data access is encouraged and those interested should contact RS (robert.stewart@kcl.ac.uk), CRIS academic lead.

For peer review only

Abstract

Objective: To determine whether gender differences in symptom presentation at first episode of psychosis remain even when controlling for substance use, age and ethnicity, using natural language processing (NLP) applied to electronic health records (EHRs).

Design, Setting, Participants: Data were extracted from EHRs of 3,350 people (62% male) who had presented to the South London and Maudsley (SLaM) NHS Trust with a first episode of psychosis between 1 April 2007 and 31 March 2017. Logistic regression was used to examine gender differences in the presentation of positive, negative, depressive, mania and disorganization symptoms.

Exposure(s) (for observational studies): Gender (male vs female)

Main Outcome(s) and Measure(s): Presence of positive, negative, depressive, mania and disorganization symptoms at initial clinical presentation

Results: Eight symptoms were significantly more prevalent in males (poverty of thought, negative symptoms, social withdrawal, poverty of speech, aggression, grandiosity, paranoia and agitation). Conversely, tearfulness, low energy, reduced appetite, low mood, pressured speech, mood instability, flight of ideas, guilt, mutism, insomnia, poor concentration, tangentiality and elation were more prevalent in females than males. Negative symptoms were more common among males (OR 1.85, 95% CI 1.33 to 2.62) and depressive and manic symptoms more common among females (0.30, 0.26 to 0.35). After adjustment for illicit substance use, the strength of associations between gender and negative, manic and depression symptoms increased, whereas gender differences in aggression, agitation, paranoia and grandiosity became insignificant.

Conclusions: There are clear gender differences in the clinical presentation of FEP. Our findings suggest that gender can have a substantial influence on the nature of clinical presentation in people with psychosis, and that this is only partly explained by exposure to illicit substance use.

For peer review only

For peer review only - http://bmjopen.bmj.com/site/about/guidelines.xhtml

Strengths and limitations

- Our methodology using natural language processing (NLP) on an electronic case register allows us to obtain the largest sample size to date in this field
- This is the first study to control for onset age, ethnicity and substance use in gender differences in symptom presentation in first episode psychosis
- By examining patients at first presentation, our findings represent differences early on in the trajectory of psychosis that are less likely to be affected by treatment.
- NLP techniques are associated with a degree of measurement error, which could impact the recorded frequencies for each symptom
- Symptoms reported in electronic health records might be impacted by gender biases among clinicians

For peer review only

Introduction

The incidence and clinical presentation of first episode psychosis (FEP) varies by gender.[1] Some of these differences, such as earlier age of onset for males, and a bimodal age distribution for females, are well established.[2–4] Others, such as symptoms at FEP presentation, are less well established.

Previous studies report that male patients with FEP present with greater negative and disorganization symptom burden and greater illicit substance use than females.[5] Female patients conversely appear to present with more affective symptoms and parasuicidal behavior than males, with affective symptoms increasing risk for parasuicidal behavior.[6,7] Despite these findings, several studies have failed to find significant clinical differences in symptoms at all.[8,9]

At population level, men show greater risk for substance use than women,[10] and this pattern is especially prominent in psychosis samples.[11–13] Negative symptoms, such as motivational deficits, are common to both psychosis and substance use disorders;[14] increased prevalence of negative symptoms in males could therefore plausibly be associated with illicit substance misuse.[3] However, no published studies have attempted to control for illicit substance use at the point of an emerging psychotic disorder.

Few studies investigating variation in psychotic symptoms at first presentation control for ethnicity. Both psychosis risk and symptomatology show ethnic variability;[1,15–17] furthermore, specific genders and ethnic groups are associated with unique and shared risk factors for psychosis.[18] The way in which these factors might interact in psychosis is unknown.[19]

Quantifying the impact of gender on symptom expression can provide a clue as to the underlying mechanisms. For example, negative symptoms are difficult to treat and correlate with poorer outcomes.[20] The cause of negative symptoms remains unknown, and this is the rate-limiting obstacle to developing new treatments. If these are genuinely more common in men, even factoring in increased substance use in males, it suggests that gender-related biological factors may play a role in their pathophysiology.[3] For example, sex-specific hypothalamic-pituitary-gonadal dysfunction are implicated in psychosis pathophysiology;[21] estrogens are known to modulate the dopaminergic and glutamatergic systems, both of which show aberrant functioning in psychosis.[22] More recently, central nervous system autoimmunity has been implicated in the etiology of psychosis. Around 80% of patients with Anti-N-methyl-d-aspartate (NMDA) receptor encephalitis are female whereas Anti-LGI1 and anti-CASPR2 encephalitis more frequently occur in males.[23] Understanding these gender differences might inform the development of novel, more targeted treatments for psychosis.

We sought to investigate gender differences in psychosis symptoms experienced by those presenting with FEP to a specialized Early Intervention Service using natural language processing (NLP) techniques applied to electronic health record (EHR) data. We hypothesized that there would be differences between the genders in terms of symptoms and substance use recorded, and that these might be impacted by age at onset, ethnicity and substance use. Natural language processing (NLP) techniques applied to free-text EHR

For peer review only - http://bmjopen.bmj.com/site/about/guidelines.xhtml

data can automatically classify patients who present with specific symptoms and other characteristics.[24] These techniques use machine learning to learn from human-annotated examples and are able to navigate term negation (e.g., “No thought disorder elicited”) and mentions irrelevant to that which is being measured (e.g. “His father has thought disorder”). This approach has previously been used to investigate transdiagnostic risk for psychosis,[25] the association of negative symptoms with antipsychotic treatment failure,[26] poor insight as a predictor of service use outcomes[27] and the association of cannabis use with hospital admissions[28] in people with psychotic disorders. Our sample of 3,350 patients represents the largest known sample in this field to date.

For peer review only

For peer review only - http://bmjopen.bmj.com/site/about/guidelines.xhtml

Methods

Reporting

We used the STROBE cross sectional checklist when writing our report. [29]

Study setting

Clinical data were obtained from de-identified EHRs held by the South London and Maudsley (SLaM) NHS Trust using the Clinical Record Interactive Search (CRIS) system. SLaM is one of the largest mental healthcare providers in Europe and implemented a fully electronic health record system from 2006 onwards. The Trust holds records for over 450,000 patients. Its provision includes four early intervention services in its catchment area of four boroughs (Croydon, Lambeth, Lewisham and Southwark) in southeast London with a population of around 1.2 million residents.

As EHRs provided limited structured information on symptomatology and substance use, a suite of natural language processing (NLP) tools has recently been developed within CRIS to allow conversion of unstructured free text, such as that of uploaded attachments and discharge summaries, into structured data. These yield complementary information on diagnoses, symptomatology and other patient characteristics.

The SLaM Biomedical Research Centre Case Register and CRIS have received ethical approval from the Oxfordshire Research Ethics Committee C (18/SC/0372) as an anonymized dataset for mental health research. As CRIS data are de-identified, individual consent was not sought.

Sample

Data were extracted for all individuals aged 16-65 years accepted to Early Intervention Services (EIS) for FEP within the SLaM catchment area (Lambeth Early Onset (LEO) Team, Southwark Team for Early Psychosis (STEP), Lewisham Early Intervention Service, and Croydon Outreach and Assertive Support Team (COAST)) between 1 April 2007 and 31 March 2017.[30] From 2016, the upper age limit of EIS increased from 35 to 65. The original sample contained 3,597 individuals. Individuals with no symptom data (n = 63) and missing ethnicity data (n = 70) were excluded, leaving a sample size of 3464 participants with complete covariate data and at least one documented symptom. Inspection of acceptance date distribution revealed mass uploads of backlogged records on three outlier days in April 2007 for one service; we therefore removed these individuals (n = 114) from analysis as they were not genuine first episodes.

Measures

Demographics

Structured data were extracted on age at accepted referral, gender, and ethnicity. Ethnicity was recoded as Asian, Black – African, Black – Caribbean, Black – Other, Mixed, Other and White (*Supplementary Table 1*).

For peer review only - http://bmjopen.bmj.com/site/about/guidelines.xhtml

Symptoms and substance use

CRIS offers a suite of NLP algorithms that are able to create structured data from unstructured free text through detection of key words and phrases. In brief, the algorithms are developed by applying cross-validated Support Vector Machines to a human-annotated training corpus of 'positive' (e.g. *'UDS shows cocaine'),* 'negative' (e.g. *'does not use cocaine'*) and irrelevant (e.g. *'cocaine is a highly addictive drug*') examples for each symptom and substance. Their performance is then quantified against unseen data in an iterative process of development and testing.[24] The full library of NLP algorithms available within CRIS and details of their development are provided on the CRIS website.[31]

These algorithms were used to extract data for 42 symptom constructs and 4 substance use indicators mentioned in the period three months either side of the date of each patient's acceptance to an EIS clinical team. Dichotomous variables were created indicating presence or absence of at least one mention of a given symptom or substance use indicator. We excluded symptoms of low prevalence (less than 0.05% of the sample). Symptoms were classified into the following domains identified in a principal axis factor analysis by Demjaha and colleagues[32] using the approach employed by Jackson *et al* [24] (see *Supplementary Table 2*): (1) positive, (2) negative, (3) disorganization, (4) manic and (5) depressive symptoms. *Insight* was assigned to (6) other. Extracted substance data concerned cocaine, cannabis, amphetamine and MDMA.

NLP algorithm performance is mainly measured using precision (proportion of relevant instances among retrieved instances) and recall (proportion of relevant instances retrieved from all relevant instances in data). Precision estimates generated through manual validation of each symptom (0.64-0.99, mean = 0.86) and substance (0.87-0.97, mean = 0.92) NLP algorithms are supplied in *Supplementary Table 3*.[24]

Diagnosis

We recorded each patient's ICD-10 diagnosis closest to 12 months from the EIS accepted date. Patient diagnoses were derived from either a structured Primary Diagnosis field or unstructured free text using NLP. Psychosis diagnoses were recoded as bipolar disorder, drug-induced psychosis, schizophrenia, psychotic depression, schizoaffective disorder or other psychosis (*Supplementary Table 4*).

Statistical analysis

All analyses were conducted using R version 3.6.0. We obtained descriptive statistics for demographic characteristics, diagnosis at 12 months, and symptom and substance use distributions.

Binary logistic regression was used to assess gender differences in symptomatology and substance use. Subsequent multivariable models controlling for age and ethnicity were constructed to examine potential confounding effects in differences in symptoms or substance use. The reference group for ethnicity was 'White', which comprised the sample majority. P-values were adjusted for multiple testing using the Benjamini-Hochberg correction (false discovery rate [FDR])[33] using the *p.adjust* package in R.

For peer review only - http://bmjopen.bmj.com/site/about/guidelines.xhtml

The adjusted models were then updated to include illicit substance use as an additional covariate. A dichotomous variable was created to flag any mention of cannabis, cocaine, MDMA or amphetamine as *present/absent*.

Given that *p*-values for male gender increased when controlling for substance use, we ran post-hoc analyses in which we (i) ran diagnostic tests for multicollinearity by calculating the variance inflation factor (VIF) for each predictor in each model, and (ii) built an additional model inclusive of a gender*substance use interaction term.

Multinomial logistic regression, implemented via the *nnet* package, was used to investigate gender differences in diagnosis at 12 months from the patient's first accepted referral to an EIS. We controlled for age and ethnicity.

Patient and public involvement

Project applications requiring access to CRIS data are subject to approval from the CRIS Oversight committee, which is service user led.

For peer review only

Results

Table 1 presents sample characteristics. The final sample comprised 3,350 patients of predominantly male gender (*n* = 2077, 62%). Median (IQR) age was 24 (20-29). White (*n* = 1037), Black-Other (*n* = 847) and Black-Caribbean (*n* = 635) were the most frequently represented ethnic groups.

<< Table 1 >>

Age distribution by gender and by substance use are presented in Figure 1. Number of referrals sharply inclines in the late teenage years for both genders. Both genders experience a sharp peak in incidence in the early twenties which falls at a greater rate for male patients than female patients. Individual density plots showing referral numbers by age stratified by amphetamine, cannabis, cocaine and MDMA use are provided in *Supplementary Figure 1.* The age by cannabis use plot most closely mirrors the distribution of overall substance use shown below. Referrals for cannabis and amphetamine users show inversed patterns around age 30.

<< Figure 1 >>

Figure 2 presents gender differences in symptomatology and substance use obtained via binary logistic regressions before and after adjustment for onset age, ethnicity and illicit substance use. Odds ratios (OR) refer to male gender as the reference group. All values and overall odds ratios for each symptom group are provided in *Supplementary Tables 5* and *6*.

Of 42 symptoms investigated, 8 were more prevalent in men, and 14 more prevalent in women before adjustment for age, ethnicity and substance use. Highly significant gender differences ($p \leq 0.001$) existed for poor appetite, low energy, tearfulness, guilt, mutism, grandiosity, negative symptoms, social withdrawal, aggression, mood instability and pressured speech. Symptoms more strongly associated with male gender included (in order of decreasing strength of association) poverty of thought, negative symptoms (general), social withdrawal, poverty of speech, aggression, grandiosity, paranoia and agitation. Male gender was also significantly associated with cannabis, cocaine and amphetamine use.

Female patients were significantly more likely to have depressive and manic symptoms recorded than male patients. In order of decreasing strength, female gender was associated with tearfulness, low energy, reduced appetite, low mood, pressured speech, mood instability, guilt, mutism, insomnia, poor concentration and elation.

Delusions and auditory and visual hallucinations did not differ significantly between genders ($p > 0.05$), but olfactory, tactile and gustatory hallucinations were reported more frequently in female patients. Flight of ideas and tangential speech were more frequently recorded in females than males, but no other disorganization symptoms showed significant gender differences.

<< Figure 2 >>

For peer review only - http://bmjopen.bmj.com/site/about/guidelines.xhtml

All significant differences bar tangential speech remained after adjustment for age and ethnicity (see *Supplementary Table 4*). After further adjustment for illicit substance use, gender differences in aggression, agitation, paranoia and grandiosity became non-significant ($p > 0.05$). Significant differences in negative and depression symptoms remained; these symptoms showed increased log odds. Some previously insignificant gender differences (poor insight, disturbed sleep and irritability) became significantly more likely in females. Elation, flight of ideas and tangential speech increased in significance. Gender differences were driven by individual, rather than broad groups of, symptoms (with the exception of disorganisation; *Supplementary Table 5*). No model variables showed variance inflation factor (VIF) values above 1.08, indicating no issues with multicollinearity (*Supplementary Table 6*). Interaction effects are presented in *Supplementary Table 5*.

There were clear gender differences in diagnosis 12 months from service accepted date, which were robust when adjusting for age and ethnicity (*Supplementary Table 7*). Males were three times more likely to receive a non-schizophrenia, updated diagnosis of drug-induced psychosis than females, and more than half as likely to receive a diagnosis of bipolar disorder than females.

For peer review only - http://bmjopen.bmj.com/site/about/guidelines.xhtml

Discussion

The present study investigated gender differences in symptomatology for 3,350 patients presenting with FEP. We found clear gender differences in reported symptoms, of which almost all remained when controlling for age and ethnicity. However, controlling for illicit substance use resulted in several changes to associations of gender with reported symptoms.

In univariate analyses, most negative symptoms, and use of all substances, were reported more frequently for male patients. Female patients showed more manic (bar grandiosity) and depressive symptoms. Our results are consistent with studies that find more negative symptoms and substance use in male FEP patients, and more affective symptoms in female patients.[5,6,34] Many of these differences are found in the general population, such as increased affective symptoms in women, and substance abuse in men.[10,35] The findings for disorganization symptoms were less clear. Where these existed, they tended towards female patients, in contrast to the findings of Thorup and colleagues.[5]

Strikingly, all significant gender differences (bar tangential speech) remained after controlling for age and ethnicity. This suggests that gender differences in symptoms at presentation are independent of age and ethnicity. To our best knowledge, no studies have accounted for ethnicity in analyses of gender symptom differences,[5,11,12,36] despite previous research indicating a significant association between ethnicity and risk of psychosis.[37] The lack of effect of referral age on gender differences is consistent with other studies' findings.[6,34,36]

After adjusting for illicit substance use, age and ethnicity, negative symptoms were still more prominent in males; manic and depressive symptoms became even more prominent in females. This suggests that gender differences in these three symptom domains reflect genuine sex differences in pathophysiology. Biologically, endocrine variation (particularly hypothalamic-pituitary-gonadal dysfunction in both sexes) has been implicated in gender differences of psychosis presentation. For example, converging epidemiological, clinical and animal research lends support to a neuroprotective effect of estrogens in women as a buffer against illness development and severity.[38] Interestingly, symptom severity appears to increase in the low estrogen phase of the menstrual cycle and during the menopause. Estrogen's neuroprotective effects have been attributed to its effects on the dopaminergic and glutamatergic systems, which are both implicated in psychosis.[21] Anatomically, disrupted sexual dimorphism of several regions in the brain has been repeatedly observed in psychosis.[3] Men and women are also differentially exposed to psychosocial risk factors for psychosis (recently summarized in an umbrella review)[1]. The immune system also varies by gender, and autoimmune central nervous system disorders that show associations with psychosis vary in incidence between men and women.[23]

While referral numbers in cannabis users peak at age 21, those in amphetamine users have bimodal peaks at ages 22 and 29. As our sample already have psychosis at first assessment, any inferences drawn from these findings are speculative. It is possible that patients with cannabis use are presenting with psychosis at a younger age, which is consistently replicated in the literature.[39] This finding could also reflect differential

For peer review only - http://bmjopen.bmj.com/site/about/guidelines.xhtml

age trends for usage of amphetamines and cannabis, with amphetamine more popularly used at a later age than cannabis. Conversely, both drugs might be used at comparable rates by both age groups, with amphetamine use demonstrating a larger effect in older age groups. It could also be that cannabis use confers the biggest immediate risk to the adolescent brain whereas amphetamine use has longer-term effects.

Our findings suggest that substance use plays an important role in clinical presentation of some symptoms, with differential effects between genders. Gender differences in aggression, agitation, paranoia and grandiosity all became insignificant, which might be explained by their associations with illicit substance use. This suggests that clinicians should take the role of illicit substance use into account when identifying strategies for symptomatic relief. In general, there is little research available into the ways in which substance use affect FEP presentation at the symptom level. Consistent with previous findings, male FEP patients were far more likely to use cannabis, cocaine and amphetamine than female FEP patients.[35] Comorbid substance use in these populations is associated with medication non-compliance, greater risk of relapse, higher suicidality and poorer outcomes in symptoms and functioning overall.[40–42]

Introduction of illicit substance use as a covariate caused the pattern of observed gender differences to change considerably, often inflating significance. Model VIF values close to 1 discount multicollinearity as a plausible mechanism for inflated probability values. One Danish study has provided initial evidence of a role for substance use in gender differences in clinical presentation in 578 patients assessed five years on from first episode.[5] The authors found significant associations between both male gender and substance abuse for negative and disorganization symptoms; controlling for substance use reduced effect sizes for male gender and removed prior effects of male gender on global assessment of functioning (GAF) symptom score.

In general, the impact of gender on clinical presentation and outcomes in first episode psychosis is under-researched. While 45,944 papers were published on psychotic disorders between 1995 and 2018, only 291 (0.01%) specifically investigated gender differences (Figure 3).

<< Figure 3 >>

This reflects other areas of medical research where the importance of gender differences are increasingly recognized, such as cardiovascular disease,[43] where clinical presentation and symptoms vary by gender leading to underdiagnosis of heart disease and increased mortality in female patients.[44] Our findings highlight the need to investigate the impact of gender differences on clinical presentation and their underlying pathophysiology, which could affect treatment outcomes following onset of FEP.

Strengths and limitations

There are some limitations to our approach. Symptom NLP algorithms vary in precision and recall performance; this variability in NLP accuracy could impact the recorded frequencies of each symptom.[24,28] However, one would expect measurement error to be spread equally across genders. Furthermore, the

For peer review only - http://bmjopen.bmj.com/site/about/guidelines.xhtml

algorithms cannot be used to infer symptom severity, as patients who are more unwell or represent complex cases typically have more documentation. Therefore gender differences in symptoms may not generalize across the range of disease severity. There was no significant difference in document count between genders, therefore this is unlikely to bias results. We had no healthy control group and therefore cannot establish whether gendered symptom differences are specific to FEP or extend to the general population. Our methodology is also unable to identify the extent to which the gender differences observed represent sex differences at the biological level. Given service changes in 2016, there also is only one year of data available for individuals aged 36 and older. Finally, symptoms reported in EHRs might to some extent reflect gender biases in recording or patients' willingness to disclose.

Despite these limitations, our approach also has its strengths. To our knowledge, this is the largest study to investigate gender differences in FEP, and the only study to control for ethnicity and substance use in doing so. By examining patients at first presentation, our findings represent differences early on in the trajectory of psychosis that are less likely to be affected by treatment.

Conclusion

We found that the clinical presentation of FEP varies by gender and that this is only partly explained by exposure to illicit substances. Most research on gender differences in psychosis has focused on candidate pathways to psychosis as a diagnostic construct. Studies are now urgently needed to link biological and psychosocial risk factors to specific symptoms so that novel, more targeted treatments can be developed.

For peer review only

For peer review only - http://bmjopen.bmj.com/site/about/guidelines.xhtml

References

1. Radua J, Ramella-Cravaro V, Ioannidis JPA, et al. What causes psychosis? An umbrella review of risk and protective factors. *World Psychiatry*. 2018;17(1):49-66. doi:10.1002/wps.20490

2. Kirkbride JB, Errazuriz A, Croudace TJ, et al. Incidence of Schizophrenia and Other Psychoses in England, 1950–2009: A Systematic Review and Meta-Analyses. Scott JG, ed. *PLoS One*. 2012;7(3):e31660. doi:10.1371/journal.pone.0031660

3. Riecher-Rössler A, Butler S, Kulkarni J. Sex and gender differences in schizophrenic psychoses—a critical review. *Arch Womens Ment Health*. 2018;21(6):627-648. doi:10.1007/s00737-018-0847-9

4. Ochoa S, Usall J, Cobo J, Labad X, Kulkarni J. Gender differences in schizophrenia and first-episode psychosis: a comprehensive literature review. *Schizophr Res Treatment*. 2012;2012:916198. doi:10.1155/2012/916198

5. Thorup A, Albert N, Bertelsen M, et al. Gender differences in first-episode psychosis at 5-year follow-up – two different courses of disease? Results from the OPUS study at 5-year follow-up. *Eur Psychiatry*. 2014;29(1):44-51. doi:10.1016/J.EURPSY.2012.11.005

6. Barajas A, Ochoa S, Obiols JE, Lalucat-Jo L. Gender Differences in Individuals at High-Risk of Psychosis: A Comprehensive Literature Review. *Sci World J*. 2015;2015:1-13. doi:10.1155/2015/430735

7. Tseliou F, Johnson S, Major B, et al. Gender differences in one-year outcomes of first-presentation psychosis patients in inner-city UK Early Intervention Services. *Early Interv Psychiatry*. 2017;11(3):215-223. doi:10.1111/eip.12235

8. Larsen TK, McGlashan TH, Johannessen JO, Vibe-Hansen L. First-episode Schizophrenia: II. Premorbid Patterns by Gender. *Schizophr Bull*. 1996;22(2):257-269. doi:10.1093/schbul/22.2.257

9. Hayashi N, Igarashi Y, Yamashina M, Suda K. *Is There a Gender Difference in a Factorial Structure of the Positive and Negative Syndrome Scale? A Test by Structural Equation Modeling*.; 2002. www.karger.comwww.karger.com/journals/psp. Accessed June 3, 2019.

10. Seedat S, Scott KM, Angermeyer MC, et al. Cross-national associations between gender and mental disorders in the World Health Organization World Mental Health Surveys. *Arch Gen Psychiatry*. 2009. doi:10.1001/archgenpsychiatry.2009.36

11. Cotton SM, Lambert M, Schimmelmann BG, et al. Gender differences in premorbid, entry, treatment, and outcome characteristics in a treated epidemiological sample of 661 patients with first episode psychosis. *Schizophr Res*. 2009;114(1-3):17-24. doi:10.1016/J.SCHRES.2009.07.002

12. Køster A, Lajer M, Lindhardt A, Rosenbaum B. Gender differences in first episode psychosis. *Soc Psychiatry Psychiatr Epidemiol*. 2008;43(12):940-946. doi:10.1007/s00127-008-0384-3

13. Talamo A, Centorrino F, Tondo L, Dimitri A, Hennen J, Baldessarini RJ. Comorbid substance-use in schizophrenia: Relation to positive and negative symptoms. *Schizophr Res*. 2006. doi:10.1016/j.schres.2006.04.004

14. Solowij N, Michie PT. Cannabis and cognitive dysfunction: Parallels with endophenotypes of schizophrenia? *J Psychiatry Neurosci*. 2007.

15. Heuvelman H, Nazroo J, Rai D. Investigating ethnic variations in reporting of psychotic symptoms: a multiple-

For peer review only - http://bmjopen.bmj.com/site/about/guidelines.xhtml

group confirmatory factor analysis of the Psychosis Screening Questionnaire. *Psychol Med*. 2018;48(16):2757-2765. doi:10.1017/S0033291718000399

16. Fusar-Poli P, Tantardini M, De Simone S, et al. Deconstructing vulnerability for psychosis: Meta-analysis of environmental risk factors for psychosis in subjects at ultra high-risk. *Eur Psychiatry*. 2017;40:65-75. doi:10.1016/j.eurpsy.2016.09.003

17. Oliver D, Reilly TJ, Baccaredda Boy O, et al. What Causes the Onset of Psychosis in Individuals at Clinical High Risk? A Meta-analysis of Risk and Protective Factors. *Schizophr Bull*. June 2019. doi:10.1093/schbul/sbz039

18. Mann F, Fisher HL, Major B, et al. Ethnic variations in compulsory detention and hospital admission for psychosis across four UK Early Intervention Services. *BMC Psychiatry*. 2014. doi:10.1186/s12916-014-0181-7

19. Hankivsky O. Women's health, men's health, and gender and health: Implications of intersectionality. *Soc Sci Med*. 2012. doi:10.1016/j.socscimed.2011.11.029

20. Downs J, Dean H, Lechler S, et al. Negative Symptoms in Early-Onset Psychosis and Their Association With Antipsychotic Treatment Failure. *Schizophr Bull*. 2019;45(1):69-79. doi:10.1093/schbul/sbx197

21. Riecher-Rössler A. Oestrogens, prolactin, hypothalamic-pituitary-gonadal axis, and schizophrenic psychoses. *The Lancet Psychiatry*. 2017. doi:10.1016/S2215-0366(16)30379-0

22. Riecher-Rössler A, Kulkarni J. Estrogens and Gonadal Function in Schizophrenia and Related Psychoses. In: Springer, Berlin, Heidelberg; 2010:155-171. doi:10.1007/7854_2010_100

23. Gold SM, Willing A, Leypoldt F, Paul F, Friese MA. Sex differences in autoimmune disorders of the central nervous system. *Semin Immunopathol*. 2019;41(2):177-188. doi:10.1007/s00281-018-0723-8

24. Jackson RG, Patel R, Jayatilleke N, et al. Natural language processing to extract symptoms of severe mental illness from clinical text: the Clinical Record Interactive Search Comprehensive Data Extraction (CRIS-CODE) project. *BMJ Open*. 2017;7(1):e012012. doi:10.1136/bmjopen-2016-012012

25. Irving J, Patel R, Oliver D, et al. Using Natural Language Processing on Electronic Health Records to Enhance Detection and Prediction of Psychosis Risk. *Schizophr Bull*. October 2020. doi:10.1093/schbul/sbaa126

26. Downs J, Dean H, Lechler S, et al. Negative Symptoms in Early-Onset Psychosis and Their Association With Antipsychotic Treatment Failure. *Schizophr Bull*. January 2018. doi:10.1093/schbul/sbx197

27. Ramu N, Kolliakou A, Sanyal J, Patel R, Stewart R. Recorded poor insight as a predictor of service use outcomes: Cohort study of patients with first-episode psychosis in a large mental healthcare database. *BMJ Open*. 2019. doi:10.1136/bmjopen-2019-028929

28. Patel R, Jayatilleke N, Broadbent M, et al. Negative symptoms in schizophrenia: a study in a large clinical sample of patients using a novel automated method. *BMJ Open*. 2015;5(9):e007619. doi:10.1136/bmjopen-2015-007619

29. von Elm E, Altman DG, Egger M, Pocock SJ, Gøtzsche PC, Vandenbroucke JP. The strengthening the reporting of observational studies in epidemiology (STROBE) statement: Guidelines for reporting observational studies. *Int J Surg*. 2014. doi:10.1016/j.ijsu.2014.07.013

30. Fusar-Poli P, Lai S, Di Forti M, et al. Early Intervention Services for First Episode of Psychosis in South London

For peer review only - http://bmjopen.bmj.com/site/about/guidelines.xhtml

and the Maudsley (SLaM): 20 Years of Care and Research for Young People. *Front Psychiatry*. 2020;11:577110. doi:10.3389/fpsyt.2020.577110

31. CRIS Natural Language Processing Applications Library. https://www.maudsleybrc.nihr.ac.uk/facilities/clinical-record-interactive-search-cris/cris-natural-language-processing/. Accessed July 23, 2020.

32. Demjaha A, Morgan K, Morgan C, et al. Combining dimensional and categorical representation of psychosis: The way forward for DSM-V and ICD-11? *Psychol Med*. 2009. doi:10.1017/S0033291709990651

33. Benjamini Y, Hochberg Y. Controlling the False Discovery Rate: A Practical and Powerful Approach to Multiple Testing. *J R Stat Soc Ser B*. 1995. doi:10.1111/j.2517-6161.1995.tb02031.x

34. Køster A, Lajer M, Lindhardt A, Rosenbaum B. Gender differences in first episode psychosis. *Soc Psychiatry Psychiatr Epidemiol*. 2008;43(12):940-946. doi:10.1007/s00127-008-0384-3

35. Larsen TK, Melle I, Auestad B, et al. Substance abuse in first-episode non-affective psychosis. *Schizophr Res*. 2006. doi:10.1016/j.schres.2006.07.018

36. Thorup A, Petersen L, Jeppesen P, et al. Gender differences in young adults with first-episode schizophrenia spectrum disorders at baseline in the Danish OPUS study. *J Nerv Ment Dis*. 2007;195(5):396-405. doi:10.1097/01.nmd.0000253784.59708.dd

37. Boydell J, van Os J, McKenzie K, et al. Incidence of schizophrenia in ethnic minorities in London: ecological study into interactions with environment. *BMJ*. 2001;323(7325):1336-1338. doi:10.1136/bmj.323.7325.1336

38. Gogos A, Sbisa AM, Sun J, Gibbons A, Udawela M, Dean B. A Role for Estrogen in Schizophrenia: Clinical and Preclinical Findings. *Int J Endocrinol*. 2015;2015:615356. doi:10.1155/2015/615356

39. Large M, Sharma S, Compton MT, Slade T, Nielssen O. Cannabis Use and Earlier Onset of Psychosis. *Arch Gen Psychiatry*. 2011;68(6):555. doi:10.1001/archgenpsychiatry.2011.5

40. Lambert M, Conus P, Lubman DI, et al. The impact of substance use disorders on clinical outcome in 643 patients with first-episode psychosis. *Acta Psychiatr Scand*. 2005. doi:10.1111/j.1600-0447.2005.00554.x

41. Cantor-Graae E, Nordström LG, McNeil TF. Substance abuse in schizophrenia: A review of the literature and a study of correlates in Sweden. *Schizophr Res*. 2001. doi:10.1016/S0920-9964(00)00114-6

42. Sorbara F, Liraud F, Assens F, Abalan F, Verdoux H. Substance use and the course of early psychosis: A 2-year follow-up of first-admitted subjects. *Eur Psychiatry*. 2003. doi:10.1016/S0924-9338(03)00027-0

43. Leonard EA, Marshall RJ. Cardiovascular Disease in Women. *Prim Care - Clin Off Pract*. 2018. doi:10.1016/j.pop.2017.10.004

44. Mosca L, Barrett-Connor E, Kass Wenger N. Sex/gender differences in cardiovascular disease prevention: What a difference a decade makes. *Circulation*. 2011. doi:10.1161/CIRCULATIONAHA.110.968792

For peer review only

For peer review only - http://bmjopen.bmj.com/site/about/guidelines.xhtml

Table 1: Sample characteristics

Characteristic	n	Male	Female
N (%)	3350	2092 (62%)	1258 (38%)
Age at referral (Median, IQR)	24 (20-29)	23 (20-28)	25 (21-30)
Ethnicity (N, %)			
Asian	234	144 (7%)	90 (7%)
Black African	635	383 (30%)	252 (20%)
Black Caribbean	216	123 (6%)	93 (7%)
Black Other	847	533 (3%)	314 (25%)
White	1037	652 (49%)	385 (31%)
Other	381	257 (12%)	124 (10%)
Early Intervention Team			
Lambeth	1084	683 (33%)	401 (32%)
Southwark	826	520 (25%)	306 (24%)
Lewisham	678	400 (19%)	278 (22%)
Croydon	761	495 (24%)	266 (21%)

Figure legends:

Figure 1. These density plots present age at admission by gender (left) and substance use (right). Density plots – a variation of the histogram – present the distribution of numerical data but with kernel smoothing to reduce the effect of noise in the data, thereby creating a smoother line. The peaks of a density plot indicate where values are concentrated.

Figure 2. Odds ratios greater than 1 indicate that a symptom is more likely to be observed in males than females, and vice versa for odds ratios less than 1. Dashed lines indicate odds ratios and confidence intervals before adjustment for covariates, with solid lines indicating estimates after adjustment.

Figure 3. While interest in psychosis has grown over time, few studies have examined the role of gender, and scarcely any have examined gender and ethnicity together.

For peer review only

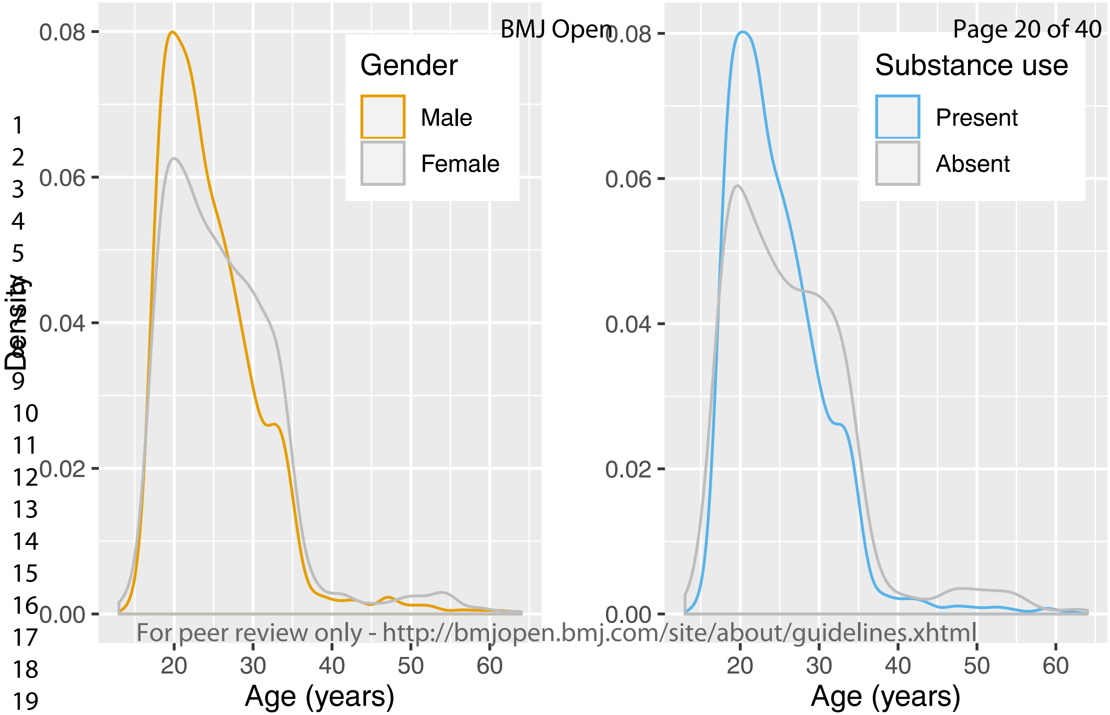

For peer review only - http://bmjopen.bmj.com/site/about/guidelines.xhtml

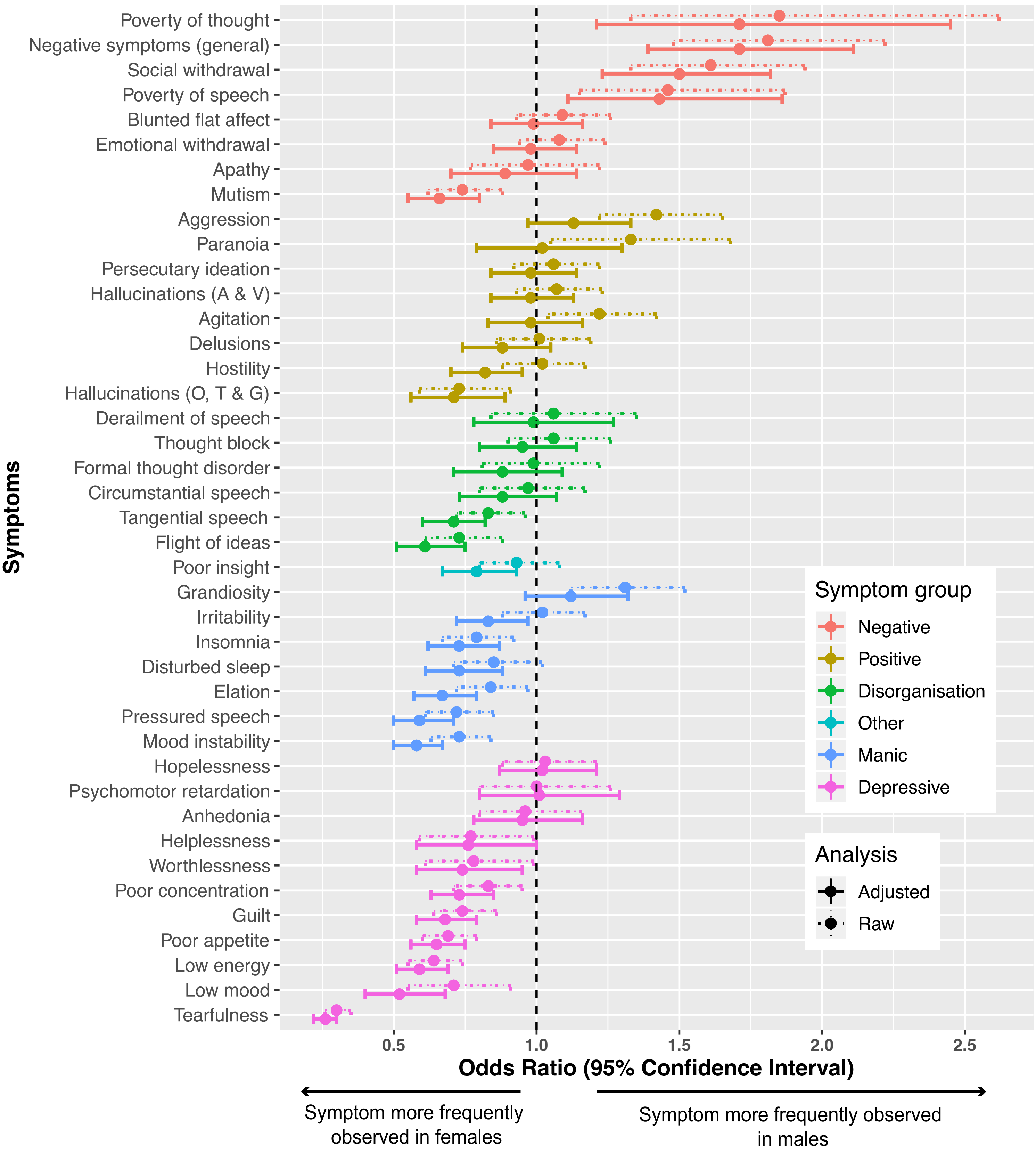
Poverty of thought
Negative symptoms (general)
Social withdrawal
Poverty of speech
Blunted flat affect
Emotional withdrawal
Apathy
Mutism
Aggression
Paranoia
Persecutary ideation
Hallucinations (A & V)
Agitation
Delusions
Hostility
Hallucinations (O, T & G)
Derailment of speech
Thought block
Formal thought disorder
Circumstantial speech
Tangential speech
Flight of ideas
Poor insight
Grandiosity
Irritability
Insomnia
Disturbed sleep
Elation
Pressured speech
Mood instability
Hopelessness
Psychomotor retardation
Anhedonia
Helplessness
Worthlessness
Poor concentration
Guilt
Poor appetite
Low energy
Low mood
Tearfulness
Symptoms
0.5
1.0
1.5
2.0
2.5
Odds Ratio (95% Confidence Interval)
Symptom group
Negative
Positive
Disorganisation
Other
Manic
Depressive
Analysis
Adjusted
Raw
Symptom more frequently observed in females
Symptom more frequently observed in males

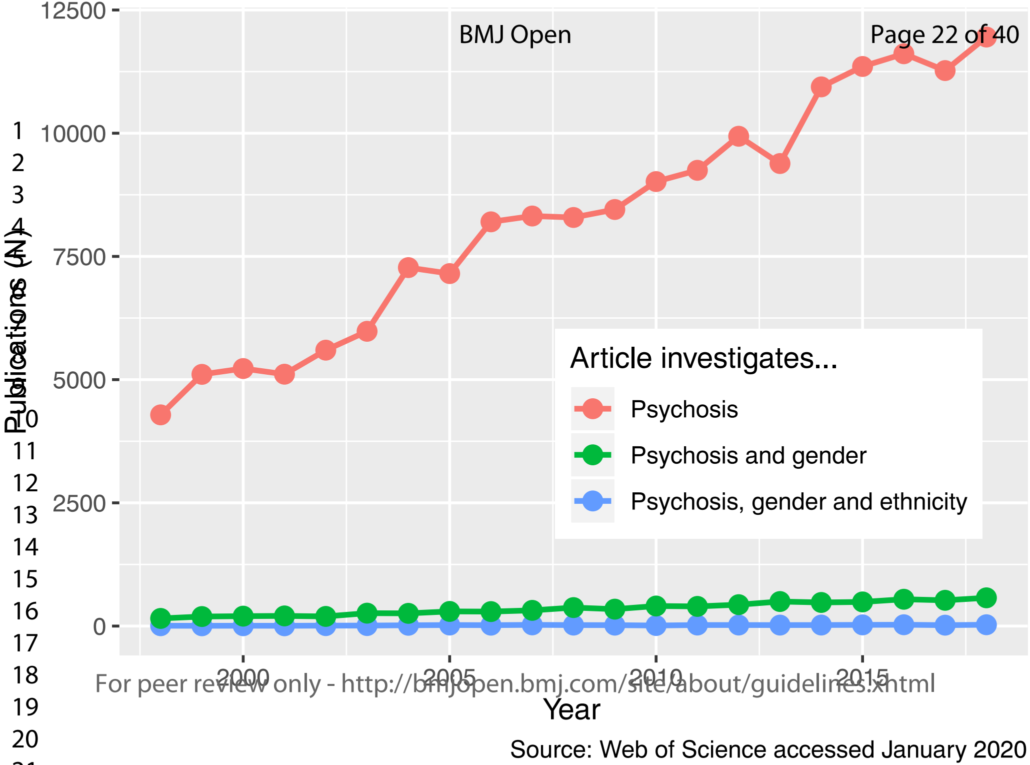

For peer review only - http://bmjopen.bmj.com/site/about/guidelines.xhtml

Supplementary material

Contents

Table 1: Predictor definitions: self-assigned ethnicity 2

Table 2: Extraction and definition of diagnoses at 12 months 3

Table 3: Manual validation of symptom and substance NLP tools 5

Figure 1: Density plots of substance use by age of onset for four different drugs: amphetamine, cannabis, cocaine and MDMA 7

Table 4: Full table of gender differences in symptom presentation and substance use 8

Table 5: VIF values for each linear model 12

For peer review only

Table 1: Predictor definitions: self-assigned ethnicity

Ethnic group	Self-assigned ethnicity captured in electronic patient records
Asian	Asian or Asian British - Bangladeshi<br>Asian or Asian British - Indian<br>Asian or Asian British - Pakistani<br>Asian or Asian British - Any other Asian background<br>Other Ethnic Groups - Chinese
Black – African	Black or Black British - African
Black – Caribbean	Black or Black British - Caribbean
Black – Other	Black or Black British - Any other Black background<br>Mixed - White and Black African<br>Mixed - White and Black Caribbean
Mixed	Mixed - White and Asian<br>Mixed - Any other mixed background
Other	Other Ethnic Groups - Any other ethnic group
Missing	Not known<br>Not recorded

For peer review only

Table 2: Extraction and definition of diagnoses at 12 months

Diagnosis code	Recorded diagnosis (structured or NLP)
Schizophrenia	'%schizophreni%' or<br>'%scizophreni%' or<br>'%schizotyp%' or<br>'%scizotyp%' or<br>'%f20%' or<br>'%f21%' or<br>'%f22%' or<br>'%f23%' or<br>'%f24%' or<br>'%f28%' or<br>'%f29%'
Schizoaffective disorder	'%schizoaffect%' or<br>'%scizoaffect%' or<br>'%f25%'
Bipolar disorder	('%f30%' or<br>'%f31%' or<br>'%manic%' or<br>'%mania%' or<br>'%bipolar%' or<br>'%bpad%' or<br>'%affective disorder%' or<br>'%mixed affective%') **and**<br>not like '%trichotillomania%' and<br>not like '%kleptomania%'
Psychotic depression	('%psychosis%' or<br>'%psychotic%' or<br>'%with psyc%' or<br>'%f32.3%' or<br>'%f33.3%') **and**<br>('%depress%' or<br>'%f32%' or<br>'%f33%') **and**<br>not like '%without%'
Drug psychosis	('%drug%' or<br>'%alcohol%' or<br>'%opioid%' or<br>'%opiate%' or<br>'%cannabi%' or<br>'%benzo%' or<br>'%hallucinogen%' or<br>'%cocaine%' or<br>'%cannabis%' or<br>'%f10%' or<br>'%f11%' or<br>'%f12%' or<br>'%f13%' or<br>'%f14%' or<br>'%f15%' or<br>'%f16%' or

For peer review only - http://bmjopen.bmj.com/site/about/guidelines.xhtml

	'%f17%' or<br>'%f18%' or<br>'%f19%') **and**<br>'%psychotic%'
Other psychosis	All other psychosis mentions not classified above.

For peer review only

For peer review only - http://bmjopen.bmj.com/site/about/guidelines.xhtml

Table 3: Manual validation of symptom and substance NLP tools

Symptom	Cohort	Annotations validated (n)	Precision
Aggression	All patients	50	76%
Agitation	All patients	50	82%
Anhedonia	Ever had depression dx	30	87%
Apathy	Ever had depression dx	30	73%
Blunted flat affect	All patients	30	93%
Circumstantial speech	All patients	50	90%
Delusion	All patients	30	87%
Derailment	All patients	50	74%
Disturbed sleep	Ever had depression dx	50	84%
Elation	All patients	30	90%
Emotional withdrawal	All patients	50	64%
Flight of ideas	All patients	50	72%
Formal thought disorder	All patients	50	72%
Grandiosity	All patients	30	97%
Guilt	Ever had depression dx	30	93%
Hallucinations (auditory and visual)	All patients	30	87%
Hallucinations (olfactory, tactile and gustatory)	All patients	50	86%
Helplessness	Ever had depression dx	30	90%
Hopelessness	Ever had depression dx	30	87%
Hostility	All patients	30	87%
Insomnia	Ever had depression dx	50	94%
Irritability	Ever had depression dx	30	93%
Low appetite	Ever had depression dx	30	97%
Low energy	Ever had depression dx	50	76%
Low mood	Ever had depression dx	100	71%
Mood instability	All patients	50	72%
Mutism	All patients	30	93%
Negative symptoms	All patients	30	87%
Paranoia	All patients	50	82%
Persecutory ideation	All patients	30	87%
Poor concentration	Ever had depression dx	50	76%
Poor insight	All patients	30	83%

For peer review only - http://bmjopen.bmj.com/site/about/guidelines.xhtml

Poverty of speech	All patients	30	87%
Poverty of thought	All patients	30	83%
Psychomotor retardation	Ever had depression dx	30	90%
Social withdrawal	All patients	30	90%
Tangential speech	All patients	30	97%
Tearfulness	Ever had depression dx	30	99%
Thought block	All patients	30	93%
Weight loss	Ever had depression dx	30	97%
Worthlessness	Ever had depression dx	30	90%
**Substance**	**Cohort**	**Annotations validated (n)**	**Precision**
Amphetamine	All patients	30	90%
Cannabis	All patients	30	93%
Cocaine	All patients	30	97%
MDMA	All patients	30	87%

For peer review only

Figure 1: Density plots of age at referral by substance use

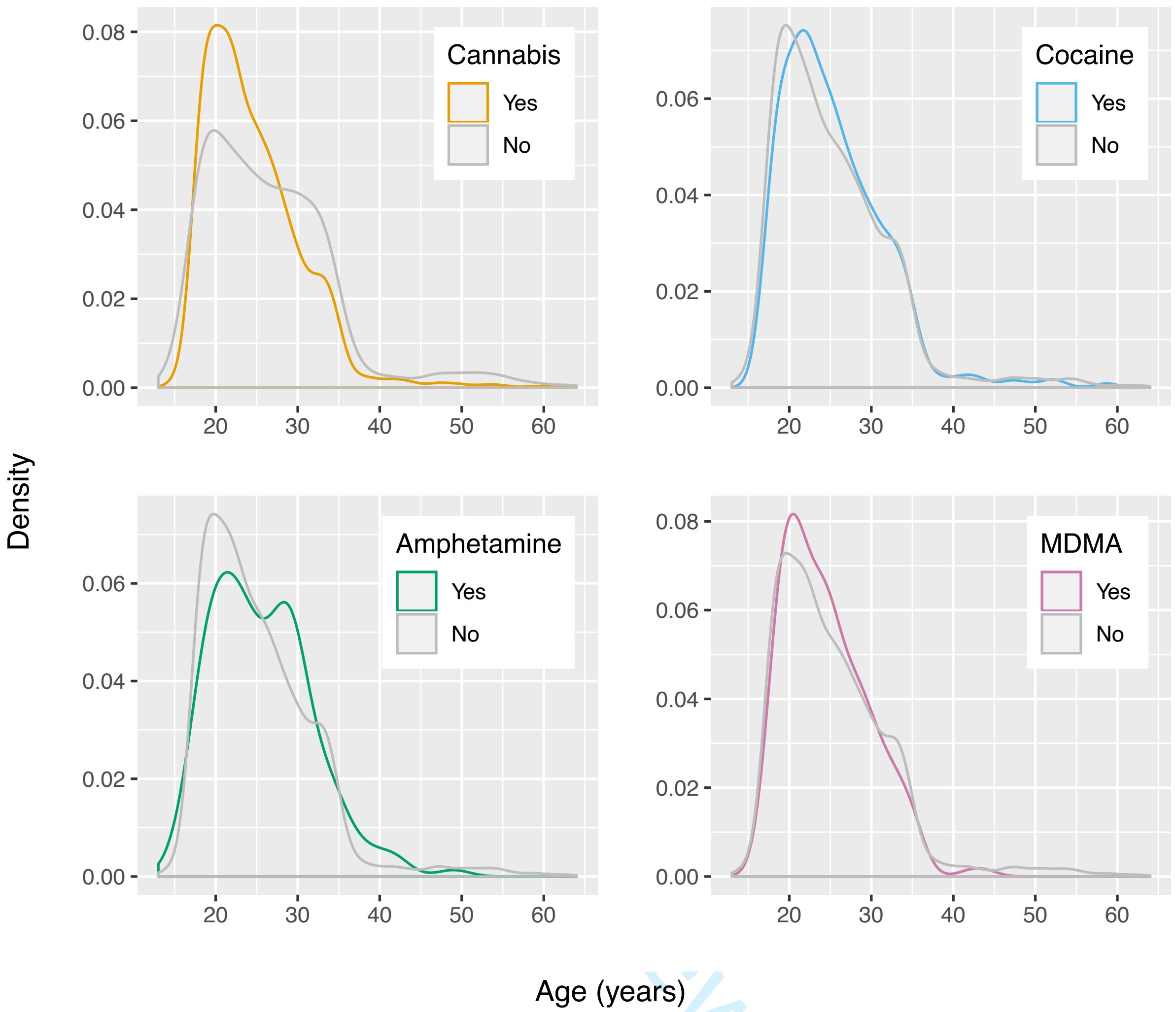

For peer review only

For peer review only - http://bmjopen.bmj.com/site/about/guidelines.xhtml

Table 4: Full table of gender differences in symptom presentation and substance use

Symptom/ Substance	N	n (%) female	n (%) male	Unadjusted		Adjusted for age and ethnicity		Adjusted for age, ethnicity and substance use		Adjusted for age, ethnicity, substance use and gender*substance use interaction	
				OR	*P value*[a]	OR	*P value*[a]	OR	*P value*[a]	OR	*P value*[a]
**Positive symptoms**											
Delusions	2584	957 (76.0)	1596 (76.3)	1.01 [0.86 - 1.19]	0.905	1.08 [0.91 - 1.27]	0.537	0.88 [0.74-1.05]	0.278	0.70 [0.54 - 0.91]	0.025*
Hallucinations (auditory and visual)	1384	503 (40.0)	869 (41.5)	1.07 [0.93 - 1.23]	0.43	1.05 [0.91 - 1.21]	0.666	0.98 [0.84-1.13]	0.854	0.97 [0.76 - 1.25]	0.909
Hallucinations (olfactory, tactile and gustatory)	376	162 (12.9)	204 (9.8)	0.73 [0.59 - 0.91]	0.007**	0.74 [0.59 - 0.92]	0.022*	0.71 [0.56-0.89]	.008**	0.76 [0.51 - 1.13]	0.336
Aggression	2318	802 (63.8)	1494 (71.4)	1.42 [1.22 - 1.65]	<0.001***	1.44 [1.24 - 1.67]	<0.001***	1.13 [0.97-1.33]	0.227	1.07 [0.84 - 1.37]	0.737
Agitation	2450	879 (69.9)	1545 (73.9)	1.22 [1.04 - 1.42]	0.017*	1.22 [1.05 - 1.43]	0.031*	0.98 [0.83-1.16]	0.903	1.01 [0.79 - 1.29]	0.996
Hostility	1282	477 (37.9)	801 (38.3)	1.02 [0.88 - 1.17]	0.858	1.03 [0.89 - 1.19]	0.839	0.82 [0.70-0.95]	0.025*	0.78 [0.59 - 1.03]	0.197
Paranoia	3092	1122 (89.2)	1917 (91.6)	1.33 [1.05 - 1.68]	0.025*	1.38 [1.09 - 1.76]	0.021*	1.02 [0.79-1.30]	0.952	0.88 [0.64 - 1.22]	0.640
Persecution ideation	1963	717 (57.0)	1221 (58.4)	1.06 [0.92 - 1.22]	0.495	1.14 [0.98 - 1.31]	0.167	0.98 [0.84-1.14]	0.865	0.89 [0.70 - 1.14]	0.544
**Negative symptoms**											
Blunted flat affect	1045	376 (29.9)	662 (31.6)	1.09 [0.93 - 1.26]	0.355	1.08 [0.93 - 1.26]	0.473	0.99 [0.84-1.16]	0.934		0.352

For peer review only

										0.83 [0.63 - 1.10]	
Emotional withdrawal	1434	520 (41.3)	903 (43.2)	1.08 [0.94 - 1.24]	0.366	1.05 [0.91 - 1.21]	0.668	0.98 [0.85-1.14]	0.896	0.91 [0.71 - 1.16]	0.629
Social withdrawal	647	185 (14.7)	454 (21.7)	1.61 [1.33 - 1.94]	<0.001***	1.56 [1.29 - 1.88]	<0.001***	1.50 [1.23-1.82]	<0.001***	1.43 [1.04 - 1.98]	0.083
Negative symptom (general)	568	150 (11.9)	411 (19.6)	1.81 [1.48 - 2.22]	<0.001***	1.76 [1.43 - 2.16]	<0.001***	1.71 [1.39-2.11]	<0.001***	1.78 [1.26 - 2.51]	0.005**
Poverty of speech	347	102 (8.1)	239 (11.4)	1.46 [1.15 - 1.87]	0.003**	1.45 [1.14 - 1.86]	0.010**	1.43 [1.11-1.86]	0.015*	1.52 [1.01 - 2.29]	0.110
Poverty of thought	187	47 (3.7)	140 (6.7)	1.85 [1.33 - 2.62]	0.001***	1.77 [1.27 - 2.52]	0.004**	1.71 [1.21-2.45]	0.009**	2.01 [1.14 - 3.64]	0.052
Apathy	343	129 (10.3)	208 (9.9)	0.97 [0.77 - 1.22]	0.815	0.97 [0.77 - 1.23]	0.89	0.89 [0.70-1.14]	0.509	0.84 [0.54 - 1.31]	0.650
Mutism	625	272 (21.6)	353 (16.9)	0.74 [0.62 - 0.88]	0.001***	0.72 [0.60 - 0.86]	0.001***	0.66 [0.55-0.80]	<0.001***	0.55 [0.39 - 0.77]	0.002 **
**Mania symptoms**											
Disturbed sleep	2733	1032 (82.0)	1664 (79.4)	0.85 [0.71 - 1.02]	0.098	0.86 [0.72 - 1.03]	0.184	0.73 [0.61 - 0.88]	0.004**	0.62 [0.47 - 0.82]	0.003**
Elation	1060	425 (33.8)	626 (29.9)	0.84 [0.72 - 0.97]	0.026*	0.82 [0.71 - 0.96]	0.033*	0.67 [0.57 - 0.79]	<0.001***	0.62 [0.46 - 0.84]	0.008 **
Grandiosity	1061	351 (27.9)	702 (33.5)	1.31 [1.12 - 1.52]	0.001***	1.33 [1.14 - 1.55]	0.001***	1.12 [0.96 - 1.32]	0.265	1.11 [0.83 - 1.49]	0.660
Insomnia	830	345 (27.4)	479 (22.8)	0.79 [0.67 - 0.92]	0.005**	0.78 [0.66 - 0.92]	0.009**	0.73 [0.62 - 0.87]	0.001***	0.73 [0.55 - 0.98]	0.097
Irritability	2032	754 (59.9)	1262 (60.2)	1.02 [0.88 - 1.17]	0.858	1.02 [0.88 - 1.18]	0.88	0.83 [0.72 - 0.97]	0.045*	0.83 [0.65 - 1.06]	0.278
Mood instability	1763	717 (57.0)	1029 (49.1)	0.73 [0.63 - 0.84]	<0.001***	0.71 [0.61 - 0.81]	<0.001***	<0.58 [0.50 - 0.67]	<0.001***	0.76 [0.60 - 0.97]	0.080
Pressured speech	698	302 (24.0)	388 (18.5)	0.72 [0.61 - 0.85]	<0.001***	0.73 [0.61 - 0.86]	0.001***	0.59 [0.50 - 0.71]	<0.001***	0.68 [0.47 - 0.96]	0.086

For peer review only - http://bmjopen.bmj.com/site/about/guidelines.xhtml

**Depressive symptoms**											
Worthless	306	129 (10.3)	171 (8.2)	0.78 [0.61 - 0.99]	0.053	0.78 [0.61 - 0.99]	0.095	0.74 [0.58 - 0.95]	0.045*	0.78 [0.49 - 1.21]	0.447
Anhedonia	546	207 (16.5)	333 (15.9)	0.96 [0.80 - 1.16]	0.746	0.97 [0.80 - 1.18]	0.854	0.95 [0.78-1.16]	0.736	0.78 [0.55 - 1.09]	0.282
Low mood	3087	1162 (92.4)	1874 (89.4)	0.71 [0.55 - 0.91]	0.011*	0.72 [0.56 - 0.93]	0.031*	0.52 [0.40-0.68]	<0.001***	0.51 [0.37 - 0.72]	0.001***
Guilt	1009	426 (33.9)	576 (27.5)	0.74 [0.64 - 0.86]	<.001***	0.74 [0.64 - 0.86]	0.001***	.68 [0.58 - 0.79]	<0.001***	0.68 [0.52 - 0.90]	0.025*
Poor concentration	2110	817 (64.9)	1265 (60.3)	0.83 [0.71 - 0.95]	0.014*	0.80 [0.69 - 0.92]	0.008**	0.73 [0.63 - 0.85]	<0.001***	0.78 [0.61 - 1.00]	0.119
Reduced appetite	1405	593 (47.1)	794 (37.9)	0.69 [0.60 - 0.79]	<0.001***	0.69 [0.60 - 0.79]	<0.001***	0.65 [0.56-0.75]	<0.001***	0.60 [0.47 - 0.77]	<0.000***
Low energy	1137	503 (40.0)	622 (29.7)	0.64 [0.55 - 0.74]	<0.001***	0.64 [0.55 - 0.74]	<0.001***	0.59 [0.51 - 0.69]	<0.001***	0.51 [0.39 - 0.67]	<0.000***
Helpless	262	113 (43.5)	147 (0.7)	0.77 [0.59 - 0.99]	0.053	0.77 [0.59 - 0.99]	0.098	0.76 [0.58 - 1.00]	0.096	0.71 [0.45 - 1.10]	0.274
Hopeless	859	311 (37.0)	529 (25.2)	1.03 [0.88 - 1.21]	0.772	1.04 [0.88 - 1.22]	0.791	1.02 [0.87-1.21]	0.864	1.07 [0.81 - 1.41]	0.810
Psychomotor retardation	357	133 (9.8)	222 (10.6)	1.00 [0.80 - 1.26]	0.971	0.99 [0.79 - 1.25]	0.972	1.01 [0.80-1.29]	0.955	0.94 [0.64 - 1.37]	0.868
Tearful	1723	864 (68.7)	833 (39.7)	0.30 [0.26 - 0.35]	<0.001***	0.30 [0.26 - 0.35]	<0.001***	0.26 [0.22-0.30]	<0.001***	0.30 [0.23 - 0.39]	<0.000***
**Disorganisation**											
Circumstantial speech	573	217 (17.2)	351 (61.8)	0.97 [0.80 - 1.17]	0.774	1.01 [0.84 - 1.22]	0.965	0.88 [0.73 - 1.07]	0.327	1.20 [0.83 - 1.72]	0.515
Derailment of speech	330	118 (9.4)	207 (9.9)	1.06 [0.84 - 1.35]	0.692	1.07 [0.84 - 1.36]	0.757	0.99 [0.78-1.27]	0.97	1.24 [0.80 - 1.92]	0.528
Flight of ideas	561	243 (19.3)	313 (14.9)	0.73 [0.61 - 0.88]	0.002**	0.74 [0.61 - 0.89]	0.005**	0.61 [0.51-0.75]	<0.001***	0.74 [0.50 - 1.09]	0.267

For peer review only - http://bmjopen.bmj.com/site/about/guidelines.xhtml

Formal thought disorder	452	169 (13.4)	279 (13.3)	0.99 [0.81 - 1.22]	0.946	1.00 [0.82 - 1.24]	0.985	0.88 [0.71-1.09]	0.368	0.87 [0.57 - 1.30]	0.688
Tangential speech	1181	471 (37.4)	693 (33.0)	0.83 [0.72 - 0.96]	0.015*	0.85 [0.73 - 0.99]	0.079	0.71 [0.60-0.82]	<0.001***	0.81 [0.61 - 1.07]	0.276
Thought block	757	272 (21.6)	475 (22.7)	1.06 [0.90 - 1.26]	0.521	1.04 [0.88 - 1.24]	0.762	0.95 [0.80-1.14]	0.722	1.09 [0.80 - 1.49]	0.751
											**Other**
Poor insight	2200	829 (65.9)	1345 (64.1)	0.93 [0.80 - 1.08]	0.405	0.98 [0.84 - 1.14]	0.883	0.79 [0.67-0.93]	0.011*	0.69 [0.54 - 0.88]	0.011*
**Substance use**											
Cocaine	958	255 (20.3)	685 (32.7)	1.91 [1.63 - 2.26]	<0.001***	1.94 [1.65 - 2.30]	<0.001***	n/a	n/a		n/a
Amphetamine	157	41 (3.3)	114 (5.4)	1.71 [1.20 - 2.49]	0.006**	1.72 [1.20 - 2.51]	0.012*	n/a	n/a		n/a
MDMA	233	79 (6.3)	149 (7.1)	1.14 [0.87 - 1.52]	0.405	1.09 [0.82 - 1.46]	0.699	n/a	n/a		n/a
Cannabis	2223	611 (48.6)	1570 (74.9)	3.18 [2.75 - 3.70]	<0.001***	3.18 [2.74 - 3.71]	<0.001***	n/a	n/a		n/a

For peer review only

Table 5: VIF values for each linear model

Model	Covariate	GVIF	Df	GVIF^(1/(2*Df))
**Appetite**	Gender	1.09	1	1.04
	Age	1.03	1	1.02
	Ethnicity	1.04	5	1.00
	Substance use	1.11	1	1.06
**Energy**	Gender	1.09	1	1.04
	Age	1.03	1	1.02
	Ethnicity	1.04	5	1.00
	Substance use	1.12	1	1.06
**Helpless**	Gender	1.08	1	1.04
	Age	1.03	1	1.02
	Ethnicity	1.04	5	1.00
	Substance use	1.11	1	1.05
**Hopeless**	Gender	1.08	1	1.04
	Age	1.03	1	1.02
	Ethnicity	1.04	5	1.00
	Substance use	1.11	1	1.05
**Psychomotor**	Gender	1.09	1	1.05
	Age	1.03	1	1.02
	Ethnicity	1.05	5	1.00
	Substance use	1.12	1	1.06
**Tearful**	Gender	1.14	1	1.07
	Age	1.03	1	1.02
	Ethnicity	1.04	5	1.00
	Substance use	1.17	1	1.08
**Weight loss**	Gender	1.09	1	1.04
	Age	1.03	1	1.02
	Ethnicity	1.04	5	1.00
	Substance use	1.11	1	1.05
**Worthless**	Gender	1.08	1	1.04
	Age	1.03	1	1.01
	Ethnicity	1.04	5	1.00
	Substance use	1.10	1	1.05
**Suicide**	Gender	1.08	1	1.04
	Age	1.03	1	1.02
	Ethnicity	1.04	5	1.00
	Substance use	1.10	1	1.05
**Anhedonia**	Gender	1.08	1	1.04
	Age	1.03	1	1.02
	Ethnicity	1.04	5	1.00
	Substance use	1.11	1	1.05
**Low Mood**	Gender	1.09	1	1.05
	Age	1.03	1	1.02
	Ethnicity	1.05	5	1.00
	Substance use	1.12	1	1.06
**Guilt**	Gender	1.09	1	1.04
	Age	1.03	1	1.02
	Ethnicity	1.04	5	1.00
	Substance use	1.11	1	1.05
**Concentration**	Gender	1.09	1	1.04
	Age	1.03	1	1.02
	Ethnicity	1.04	5	1.00
	Substance use	1.11	1	1.06
**Mutism**	Gender	1.10	1	1.05
	Age	1.03	1	1.02
	Ethnicity	1.05	5	1.00
	Substance use	1.12	1	1.06
**Circumstantial speech**	Gender	1.07	1	1.04
	Age	1.04	1	1.02
	Ethnicity	1.04	5	1.00
	Substance use	1.11	1	1.05
**Derailment of speech**	Gender	1.07	1	1.04
	Age	1.03	1	1.02
	Ethnicity	1.04	5	1.00
	Substance use	1.10	1	1.05

For peer review only - http://bmjopen.bmj.com/site/about/guidelines.xhtml

For peer review only

**Flight of ideas**	Gender	1.08	1	1.04
	Age	1.03	1	1.02
	Ethnicity	1.04	5	1.00
	Substance use	1.10	1	1.05
**Formal thought disorder**	Gender	1.08	1	1.04
	Age	1.03	1	1.02
	Ethnicity	1.04	5	1.00
	Substance use	1.10	1	1.05
**Tangential speech**	Gender	1.09	1	1.04
	Age	1.04	1	1.02
	Ethnicity	1.04	5	1.00
	Substance use	1.13	1	1.06
**Thought block**	Gender	1.08	1	1.04
	Age	1.03	1	1.01
	Ethnicity	1.04	5	1.00
	Substance use	1.10	1	1.05
**Insight**	Gender	1.09	1	1.05
	Age	1.04	1	1.02
	Ethnicity	1.06	5	1.01
	Substance use	1.14	1	1.07
**Disturbed sleep**	Gender	1.09	1	1.05
	Age	1.03	1	1.02
	Ethnicity	1.05	5	1.00
	Substance use	1.12	1	1.06
**Elation**	Gender	1.09	1	1.05
	Age	1.03	1	1.01
	Ethnicity	1.05	5	1.00
	Substance use	1.12	1	1.06
**Grandiosity**	Gender	1.07	1	1.04
	Age	1.03	1	1.02
	Ethnicity	1.05	5	1.00
	Substance use	1.10	1	1.05
**Insomnia**	Gender	1.08	1	1.04
	Age	1.03	1	1.02
	Ethnicity	1.04	5	1.00
	Substance use	1.11	1	1.05
**Irritability**	Gender	1.09	1	1.05
	Age	1.03	1	1.02
	Ethnicity	1.05	5	1.00
	Substance use	1.12	1	1.06
**Apathy**	Gender	1.08	1	1.04
	Age	1.03	1	1.02
	Ethnicity	1.04	5	1.00
	Substance use	1.10	1	1.05
**Blunted flat affect**	Gender	1.08	1	1.04
	Age	1.03	1	1.02
	Ethnicity	1.04	5	1.00
	Substance use	1.11	1	1.05
**Emotional withdrawal**	Gender	1.08	1	1.04
	Age	1.03	1	1.01
	Ethnicity	1.04	5	1.00
	Substance use	1.11	1	1.05
**Negative symptoms (general)**	Gender	1.07	1	1.04
	Age	1.02	1	1.01
	Ethnicity	1.04	5	1.00
	Substance use	1.10	1	1.05
**Poverty of speech**	Gender	1.09	1	1.04
	Age	1.03	1	1.02
	Ethnicity	1.04	5	1.00
	Substance use	1.11	1	1.05
**Poverty of thought**	Gender	1.08	1	1.04
	Age	1.02	1	1.01
	Ethnicity	1.04	5	1.00
	Substance use	1.10	1	1.05
**Social withdrawal**	Gender	1.07	1	1.04
	Age	1.02	1	1.01

For peer review only - http://bmjopen.bmj.com/site/about/guidelines.xhtml

	Ethnicity	1.04	5	1.00
	Substance use	1.10	1	1.05
**Aggression**	Gender	1.08	1	1.04
	Age	1.03	1	1.02
	Ethnicity	1.05	5	1.01
	Substance use	1.12	1	1.06
**Agitation**	Gender	1.09	1	1.04
	Age	1.03	1	1.02
	Ethnicity	1.05	5	1.01
	Substance use	1.12	1	1.06
**Delusion**	Gender	1.09	1	1.04
	Age	1.03	1	1.02
	Ethnicity	1.05	5	1.00
	Substance use	1.12	1	1.06
**Hallucinations (auditory and visual)**	Gender	1.08	1	1.04
	Age	1.03	1	1.02
	Ethnicity	1.04	5	1.00
	Substance use	1.11	1	1.05
**Hostility**	Gender	1.09	1	1.04
	Age	1.03	1	1.02
	Ethnicity	1.05	5	1.00
	Substance use	1.12	1	1.06
**Paranoia**	Gender	1.08	1	1.04
	Age	1.03	1	1.01
	Ethnicity	1.04	5	1.00
	Substance use	1.11	1	1.05
**Persecution ideation**	Gender	1.08	1	1.04
	Age	1.04	1	1.02
	Ethnicity	1.05	5	1.00
	Substance use	1.12	1	1.06
**Hallucinations (olfactory, tactile and gustatory)**	Gender	1.08	1	1.04
	Age	1.03	1	1.02
	Ethnicity	1.04	5	1.00
	Substance use	1.11	1	1.05
**Mood instability**	Gender	1.11	1	1.05
	Age	1.03	1	1.02
	Ethnicity	1.04	5	1.00
	Substance use	1.13	1	1.06
**Pressured speech**	Gender	1.09	1	1.04
	Age	1.03	1	1.02
	Ethnicity	1.04	5	1.00
	Substance use	1.11	1	1.06

For peer review only - http://bmjopen.bmj.com/site/about/guidelines.xhtml

Table 6: Gender differences in diagnosis at 12 months

Diagnosis at 12 months	N	N (%) Female	N (%) Male	Unadjusted		Adjusted for age and ethnicity	
				OR (95% CI)	*P value*[a]	OR (95% CI)	*P value*[a]
Schizophrenia	1556	519 (41%)	1037 (49%)	*Ref.*	*Ref.*	*Ref.*	*Ref.*
Bipolar disorder	165	89 (7%)	76 (4%)	0.43 [0.31 - 0.59]	<0.001***	0.41 [0.30 - 0.57]	<0.001***
Drug-induced psychosis	112	16 (1%)	96 (5%)	3.00 [1.75 - 5.15]	<0.001***	2.98 [1.73 - 5.12]	<0.001***
Psychotic depression	136	77 (6%)	59 (3%)	0.38 [0.27 - 0.55]	<0.001***	0.39 [0.27 - 0.55]	<0.001***
Schizoaffective	57	32 (3%)	25 (1%)	0.39 [0.23 - 0.66]	<0.001***	0.39 [0.23 - 0.66]	<0.001***
Other psychosis	1324	525 (42%)	799 (38%)	0.76 [0.65 - 0.89]	<0.001***	0.75 [0.64 - 0.88]	0.001

[a] P-values adjusted for multiple testing via FDR

For peer review only

Reporting checklist for cross sectional study.

Based on the STROBE cross sectional guidelines.

Instructions to authors

Complete this checklist by entering the page numbers from your manuscript where readers will find each of the items listed below.

Your article may not currently address all the items on the checklist. Please modify your text to include the missing information. If you are certain that an item does not apply, please write "n/a" and provide a short explanation.

Upload your completed checklist as an extra file when you submit to a journal.

In your methods section, say that you used the STROBE cross sectionalreporting guidelines, and cite them as:

von Elm E, Altman DG, Egger M, Pocock SJ, Gotzsche PC, Vandenbroucke JP. The Strengthening the Reporting of Observational Studies in Epidemiology (STROBE) Statement: guidelines for reporting observational studies.

		Reporting Item	Page Number
**Title and abstract**			
Title	#1a	Indicate the study’s design with a commonly used term in the title or the abstract	1
Abstract	#1b	Provide in the abstract an informative and balanced summary of what was done and what was found	4
**Introduction**			
Background / rationale	#2	Explain the scientific background and rationale for the investigation being reported	5
Objectives	#3	State specific objectives, including any prespecified hypotheses	5
**Methods**			
Study design	#4	Present key elements of study design early in the paper	7

For peer review only

Setting	#5	Describe the setting, locations, and relevant dates, including periods of recruitment, exposure, follow-up, and data collection	7
Eligibility criteria	#6a	Give the eligibility criteria, and the sources and methods of selection of participants.	7
	#7	Clearly define all outcomes, exposures, predictors, potential confounders, and effect modifiers. Give diagnostic criteria, if applicable	7-8
Data sources / measurement	#8	For each variable of interest give sources of data and details of methods of assessment (measurement). Describe comparability of assessment methods if there is more than one group. Give information separately for for exposed and unexposed groups if applicable.	7-8
Bias	#9	Describe any efforts to address potential sources of bias	8
Study size	#10	Explain how the study size was arrived at	7
Quantitative variables	#11	Explain how quantitative variables were handled in the analyses. If applicable, describe which groupings were chosen, and why	8
Statistical methods	#12a	Describe all statistical methods, including those used to control for confounding	8-9
Statistical methods	#12b	Describe any methods used to examine subgroups and interactions	8-9
Statistical methods	#12c	Explain how missing data were addressed	7
Statistical methods	#12d	If applicable, describe analytical methods taking account of sampling strategy	n/a
Statistical methods	#12e	Describe any sensitivity analyses	n/a
**Results**			
Participants	#13a	Report numbers of individuals at each stage of study—eg numbers potentially eligible, examined for eligibility, confirmed eligible, included in the study, completing follow-up, and analysed. Give information separately for for exposed and unexposed groups if applicable.	7, 10

For peer review only

Participants	#13b	Give reasons for non-participation at each stage	7
Participants	#13c	Consider use of a flow diagram	n/a
Descriptive data	#14a	Give characteristics of study participants (eg demographic, clinical, social) and information on exposures and potential confounders. Give information separately for exposed and unexposed groups if applicable.	10
Descriptive data	#14b	Indicate number of participants with missing data for each variable of interest	7
Outcome data	#15	Report numbers of outcome events or summary measures. Give information separately for exposed and unexposed groups if applicable.	8
Main results	#16a	Give unadjusted estimates and, if applicable, confounder-adjusted estimates and their precision (eg, 95% confidence interval). Make clear which confounders were adjusted for and why they were included	10-11
Main results	#16b	Report category boundaries when continuous variables were categorized	n/a
Main results	#16c	If relevant, consider translating estimates of relative risk into absolute risk for a meaningful time period	n/a
Other analyses	#17	Report other analyses done—e.g., analyses of subgroups and interactions, and sensitivity analyses	11
**Discussion**			
Key results	#18	Summarise key results with reference to study objectives	12
Limitations	#19	Discuss limitations of the study, taking into account sources of potential bias or imprecision. Discuss both direction and magnitude of any potential bias.	13
Interpretation	#20	Give a cautious overall interpretation considering objectives, limitations, multiplicity of analyses, results from similar studies, and other relevant evidence.	13-14
Generalisability	#21	Discuss the generalisability (external validity) of the study results	14

For peer review only

Other Information

Funding	#22	Give the source of funding and the role of the funders for the present study and, if applicable, for the original study on which the present article is based	2

The STROBE checklist is distributed under the terms of the Creative Commons Attribution License CC-BY. This checklist was completed on 14. July 2020 using https://www.goodreports.org/, a tool made by the EQUATOR Network in collaboration with Penelope.ai

For peer review only
